# A shear-induced limit on bacterial surface adhesion in fluid flow

**DOI:** 10.1073/pnas.2516069123

**Published:** 2026-01-21

**Authors:** Edwina F. Yeo, Benjamin J. Walker, Philip Pearce, Mohit P. Dalwadi

**Affiliations:** ^a^Department of Mathematics, University College London, London WC1H 0AY, United Kingdom; ^b^Institute for the Physics of Living Systems, Faculty of Mathematical and Physical Sciences, University College London, London WC1H 0AY, United Kingdom; ^c^Mathematical Institute, Mathematical, Physical and Life Sciences Division, University of Oxford, Oxford OX2 6GG, United Kingdom

**Keywords:** bacterial adhesion, active flow, biofilm formation

## Abstract

Controlling bacterial surface adhesion and subsequent biofilm formation in fluid systems is crucial for the safety and efficacy of medical and industrial processes. Here, we theoretically examine the transport of bacteria close to surfaces, isolating how the key processes of bacterial motility and fluid flow interact and alter surface adhesion. We exploit the disparity between the fluid velocity and the swimming velocity of common motile bacteria and, using a hybrid asymptotic-computational approach, we systematically derive the coarse-grained bacterial diffusivity close to surfaces as a function of swimming speed, rotational diffusivity, and shape. We calculate an analytical upper bound for the bacterial adhesion rate by considering the scenario in which bacteria adhere irreversibly to the surface on first contact. Our theory predicts that maximal adhesion occurs at intermediate flow rates: At lower flow rates, increasing flow increases surface adhesion, while at higher flow rates, adhesion is decreased by shear-induced cell reorientation.

## Significance

The prevention and management of bacterial contamination relies on accurately predicting the rate at which bacteria adhere to surfaces. This rate is especially challenging to predict in fluid systems, such as urinary catheters or food processing tanks, in which bacterial adhesion depends on a complex range of factors including the speed of fluid flow, the chemistry of the surface and the species of bacteria. To disentangle these effects, we use agent-based modeling and systematic mathematical theory and quantify the combined effects of flow and bacterial motility on adhesion. Our theory provides a quantitative upper bound for the bacterial adhesion rate, which explains our counterintuitive observation that bacterial adhesion is greatest at intermediate flow speeds.

## 1. Introduction

Almost two-thirds of recorded bacteria are motile: able to propel themselves in search of nutrients and adhere to surfaces (1). After attachment to a surface, bacteria can form dense colonies known as biofilms, which are the cause of the fouling of products in food processing, the contamination of drinking water supplies and more than half of all healthcare-associated infections (2, 3). In many of these industrial and natural environments, bacterial transport during the adhesion process is dominated by fluid flow. Despite intensive study, the overall effect of flow on bacterial surface attachment is not known; experimental studies have found that increasing flow rates can either increase or decrease bacterial surface colonization, depending on the bacterial species, the surface properties, and the microfluidic setup (4–9). For passive particles, such as metal or plastic nanoparticles, it is well known that higher mass transport occurs at higher flow rates, which in turn generates greater surface adhesion (10). By contrast, during bacterial surface adhesion, the biophysical cellular processes that regulate attachment (and, sometimes, subsequent detachment) are altered by flow (6). In many cases, adhesion rate per cell decreases with higher shear forces (6, 11). However, species including *Escherichia coli* and *Pseudomonas aeruginosa* have been found to exhibit increased cellular surface adhesion forces in increased shear, because of shear-sensitive catch bonds (12) and the effect of flow-induced reorientation on contact area (7), respectively. Overall, the specific contributions of cell motility, fluid transport, surface chemistry, and bacterial appendages to bacterial surface adhesion have not been identified.

As a step toward addressing this complex problem, we characterize how fluid flow affects bacterial surface attachment in the simplified scenario where bacteria adhere instantly and irreversibly with the surface upon first contact. The irreversible surface adhesion of passive particles can be predicted using an adhesion rate derived from classic Lévêque theory, also referred to as the Smoluchowski–Levich (SL) approximation (13–15). This theory predicts a particle adhesion rate of $J\left(x\right)∼0.538c_{\infty }{\left(D^{2}\dot{\gamma }/x\right)}^{1/3}$ at a downstream distance *x* from the source of the particles, where *D* is the particle diffusivity, $c_{\infty }$ is the bulk concentration of particles and $\dot{\gamma }$ is the shear rate of the flow, with the prefactor calculated using boundary layer analysis. This classic result predicts that passive particle adhesion scales with ${\dot{\gamma }}^{1/3}$ as the flow rate increases, capturing the effect of increased mass transport. However, the adhesion of active bacteria to surfaces cannot in general be described by this classic adhesion rate; bacterial dispersivity does not arise from thermal fluctuations but rather from motility, the interaction of which with fluid flow can produce enhanced nonisotropic diffusion absent from Lévêque’s analysis (14). When suspended in an otherwise quiescent fluid, the effective diffusivity of a collection of bacteria with swimming speed $V_{s}$ and rotational diffusion coefficient $D_{r}$ is isotropic with coefficient $D_{\text{quies}}\approx V_{s}^{2}/2D_{r}$ in 2D (16). The effect of combining motility and flow, in the absence of adhesion, has been analyzed using generalized Taylor dispersion theory (GTD) (17). This theory provides an effective diffusion tensor and velocity vector that are both spatially uniform (although potentially anisotropic) by averaging over local changes in orientation and position and taking the long-time limit of bacterial movement. In the high-flow environment we consider, there are large spatial gradients in cell alignment close to the surface, so the classical assumptions of spatial homogeneity required to derive GTD models break down (18, 19). This means previously calculated GTD diffusion tensors cannot be readily applied in combination with the Lévêque solution to predict bacterial adhesion in flow.

In this paper, we theoretically address this fundamental problem of bacterial surface adhesion in flow by extending Lévêque’s analysis to include the additional complexities of bacterial motility. We first consider a collection of individual bacteria using an agent-based framework, before systematically upscaling this to a continuum description of dilute active suspensions (20). Bacterial adhesion results in a diffusive boundary layer where bacterial density is depleted, in which we derive analytical solutions for both the bacterial orientation and density. We identify the maximum theoretical adhesion rate of motile bacteria to surfaces, in the absence of surface chemistry effects or imperfect adhesion events. This adhesion rate increases as the flow rate increases for low shear, reaches a maximal adhesion at intermediate shear, and beyond this the adhesion rate is reduced by shear-induced bacterial reorientation. These results can be applied to both spherical and elongated bacteria in settings where the flow rate exceeds the swimming speed, such as urinary catheters, the human gut, and slow-flowing rivers.

## 2. Results

### 2.1. Agent-Based Model for Bacterial Transport and Adhesion.

**Fig. 1.**
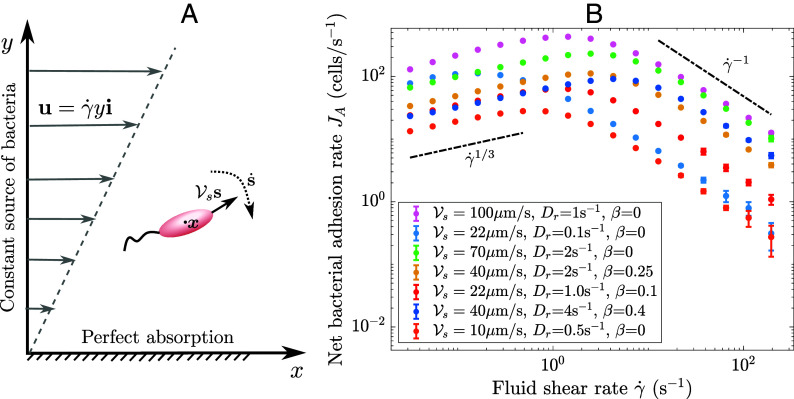
Agent-based simulations of bacterial adhesion in shear flow. (*A*) Individual bacterium transport in shear flow: Each bacterium is advected by the fluid transport u and swims with speed $V_{s}$ in direction s, which evolves according to Eq. **2**. (*B*) Net bacterial adhesion rate $J_{A}$ to a surface of length l=2.25  mm as the shear rate $\dot{\gamma }$ varies, with SD across simulations denoted via errorbars (which are very small for lower shear rates). At low shear, the adhesion rate increases as $J_{A}∼{\dot{\gamma }}^{1/3}$ and, at high shear, the adhesion rate decreases as $J_{A}∼{\dot{\gamma }}^{-1}$, with analysis of these scalings provided in *Results*.

We consider the overdamped transport of a dilute active bacterial suspension in a two-dimensional (x,y)-plane with an imposed shear flow $u=\dot{\gamma }yi$, where $\dot{\gamma }$ is the shear rate and i is the unit vector in the *x*-direction. We consider the region x,y>0, as shown in Fig. 1*A*. We model the bacteria as spheroidal, rigid bodies. Motivated by biomedical and industrial applications in which bacterial density is low, for instance in urinary catheters, in which bacterial density is less than 1% by volume (21), we neglect physical and hydrodynamic interactions between multiple bacteria. For now, we do not consider any further hydrodynamic bacteria-surface coupling or any specific chemical or biomechanical surface interactions, although such effects are investigated later (*SI Appendix*, § SVII). We assume instead that the bacteria irreversibly adhere to the surface upon first contact (ignoring any lubrication forces) in order to isolate the roles of flow and motility on adhesion. Each bacterium is described by its position vector x and unit orientation vector s, the latter evolving stochastically and capturing the random changes in orientation of the bacteria. The bacteria are advected by the fluid flow and propel themselves in the direction of their orientation vector with fixed speed $V_{s}$. Hence, each bacterium’s position is described by a Langevin equation:

[1] $$\begin{array}{c} \text{d}x=(u+V_{s}s)\;\text{d}t. \end{array}$$

Here, Brownian motion of bacteria arising from thermal effects is negligible relative to the emergent diffusion arising from swimming. For example, *E. coli* has a thermal diffusion coefficient of $D_{T}\approx$ 0.2 μm^2^ s^−1^ in comparison to an emergent diffusion coefficient in a quiescent fluid of $D_{\text{quies}}\approx$ 240 μms ^−1^ (calculated from Table 1).

**Table 1.** Bacterial motility parameter values

Name	Strain	Symbol	Units	Value	Source
Swimming speed *E. coli*	HCB437	$V_{s}$	μms ^−1^	22±5	(27)
Swimming speed *P. aeruginosa*	PAO1	$V_{s}$	μms ^−1^	23±6	(35)
Rotational diffusion coeff. *E. coli*	RP437	$D_{r}$	s ^−1^	1	(36)
Rotational diffusion coeff. *P. aeruginosa*	PAO1	$D_{r}$	s ^−1^	0.036	(37)
Bretherton param. *E. coli*	HCB437	*β*	–	0.88	(27)
Bretherton param. *P. aeruginosa*	Species averages given	*β*	–	0.88	(38)

The orientation vector s evolves as the bacteria are rotated by the fluid flow, an effect that we refer to as shear alignment, and diffuses randomly at a rate proportional to the rotational diffusion coefficient $D_{r}$. This rotational diffusion captures changes in direction owing to the bacterial swimming mechanism. Spherical bacteria experience a uniform rate of rotation in shear flow proportional to the rate of rotation tensor $W=(\nabla u-\nabla u^{T})/2$. By contrast, nonspherical bacteria in shear flow experience a nonuniform rate of rotation, resulting in the classical Jeffery orbit (22). This effect is captured by the addition of a term proportional to the rate of strain tensor $E=(\nabla u+\nabla u^{T})/2$. Hence, each bacterium’s orientation is described by an additional Langevin equation, written in vector form:

[2] $$\begin{array}{c} \text{d}s=\left(\left(\beta E+W\right)s\right)\;\text{d}t+\sqrt{2D_{r}}\;\text{d}W\times s, \end{array}$$

although we simulate dynamics restricted to the plane of the flow (we examine bacterial adhesion in 3D using the continuum model in *SI Appendix*, §SVI). The term dW is a vector Wiener process with mean zero and SD $\sqrt{\text{d}t}$. In Eq. **2**, bacterial shape is captured via the Bretherton parameter β∈[0,1), which is zero for spherical bacteria and approaches one for infinitely elongated bacteria.

The simulated net adhesion rates of different bacterial species to a surface of length *l* at different fluid flow rates are shown in Fig. 1*B* (numerical framework presented in *Materials and Methods*). We also vary the motility parameters $V_{s}$ and $D_{r}$ between species and calculate the net adhesion rate to the entire surface in cells per second as a function of fluid shear rate, removing any dependence on distance from the bacterial source *x*. The computed adhesion rates across all motility parameters increase with flow rate at low shear, with the adhesion rate appearing to increase proportionally to ${\dot{\gamma }}^{1/3}$. This agrees with the scaling predicted by Lévêque for passive particles in flow (14). However, as shear increases further, the adhesion reaches a maximum value before decreasing. The shear rate that generates maximal adhesion differs for each set of bacterial parameters. These results demonstrate that, even with perfect surface adhesion, there appears to be a maximal rate of bacterial adhesion to surfaces in fluid flow.

### 2.2. Continuum Model for Bacterial Transport and Adhesion.

To understand these observations, we upscale the individual dynamics governed by Eqs. **1** and **2** to describe the dynamics of a collection of bacteria using a continuum partial differential equation (PDE) model, which is amenable to analysis. We use the continuum active suspension model presented in ref. 20, and present the details of this upscaling procedure in *SI Appendix*, § SI. By performing a multiscale (asymptotic) analysis of the continuum PDE model near the surface, we will systematically investigate and explain why adhesion exhibits a maximum as the flow increases, and subsequently calculate the flow rate at which this maximum occurs for given bacterial species.

For ease of analysis, we nondimensionalize our model using a reference velocity scale $U=\dot{\gamma }L$ for the external flow, with reference lengthscale L that is much larger than the size of an individual bacterium, and the timescale of fluid transport ${\dot{\gamma }}^{-1}$. On the continuum scale, instead of tracking the position of each bacterium we now track three continuum quantities. These are i) the density of the collection ρ(x,t), which captures the number of bacteria per unit area. ii) The mean orientation vector, known as the polar order parameter n(x,t), which is the continuum equivalent of the orientation vector s. iii) The angular distribution of the collection through the Q(x,t) tensor, also known as the nematic order tensor. This tensor can be considered a measure of how locally ordered the bacteria are, noting that Q=0 when the angular distribution of the collection is uniform. The bacterial density evolves according to a conservation law, in the form of the following PDE:

[3] $$\begin{array}{cc} \frac{D\rho }{\mathrm{Dt}} & =-V_{s}\nabla \cdot \left(\rho n\right), \end{array}$$

where the material derivative on the left-hand side captures the advection of the bacteria by the flow and the flux term on the right-hand side captures the swimming of the collection in the direction n. The dimensionless parameter $V_{s}=V_{s}/\dot{\gamma }L$ is the ratio of the bacterial swimming speed to the flow speed. The mean orientation vector n and nematic order tensor Q capture the bacterial rotation described in Eq. **2** and are described by the following PDEs:

[4a] $$\begin{array}{cc} \frac{D(\rho n)}{\mathrm{Dt}} & =-V_{s}\left(\nabla \cdot \left(\rho Q\right)+\frac{1}{2}\nabla \rho \right)+\left(\rho In-T\right):\left(\beta E+W\right)-\frac{\rho n}{Pe_{r}}, \end{array}$$

[4b] $$\begin{array}{cc} \frac{D(\rho Q)}{\mathrm{Dt}} & =-V_{s}\left(\nabla \cdot T+\frac{I}{2}\nabla \cdot \left(\rho n\right)\right)+\beta \rho \left(E(Q+I/2)+(Q+I/2)E\right)+\rho \left(WQ-QW\right)-2\beta G:E-\frac{4\rho Q}{Pe_{r}}. \end{array}$$

On the left-hand side of both Eq. **4a** and **4b**, the material derivative captures the effect of flow advecting bacteria from upstream. On the right-hand sides: the flux terms, each premultiplied by the swimming speed $V_{s}$, capture the effect of bacterial swimming while the source terms featuring the fluid tensors W and E and the identity tensor I capture shear alignment. The final source term on the right-hand sides of both equations in Eq. **4** captures rotational diffusion, which is inversely proportional to the rotational Péclet number, $Pe_{r}=\dot{\gamma }/D_{r}$, the ratio of fluid rotation to rotational diffusion. There are two additional tensors introduced in Eq. **4**: T, which is rank three, and G, which is rank four. These tensors capture higher-order effects that arise when systematically upscaling collective behavior. We relate these tensors to the mean orientation and nematic order tensor using approximations known as closures. In 2D, these tensors have components defined in index notation as follows (23, 24):

[5a] $$\begin{array}{cc} T_{\mathrm{ijk}} & =\frac{\rho }{4}\left(\delta_{\mathrm{ij}}n_{k}+\delta_{\mathrm{ik}}n_{j}+\delta_{\mathrm{jk}}n_{i}\right), \end{array}$$

[5b] $$\begin{array}{cc} G_{\mathrm{ijkl}} & =\frac{\rho }{8}\left(\delta_{\mathrm{ij}}\delta_{\mathrm{lk}}+\delta_{\mathrm{ik}}\delta_{\mathrm{jl}}+\delta_{\mathrm{il}}\delta_{\mathrm{jk}}\right)+\frac{\rho }{6}\left(\delta_{\mathrm{ij}}Q_{\mathrm{lk}}+\delta_{\mathrm{ik}}Q_{\mathrm{jl}}+\delta_{\mathrm{il}}Q_{\mathrm{jk}}+\delta_{\mathrm{jk}}Q_{\mathrm{il}}+\delta_{\mathrm{jl}}Q_{\mathrm{ik}}+\delta_{\mathrm{jk}}Q_{\mathrm{ij}}\right), \end{array}$$

where $\delta_{\mathrm{ij}}$ is the Kronecker delta. We demonstrate that these closures, Eq. **5**, are very accurate at capturing the underlying agent-based model when: $Pe_{r}≲1$ for highly elongated swimmers β∈(0.5,1), $Pe_{r}≲10$ for modestly elongated swimmers β∈(0,0.5) and $Pe_{r}≲100$ for circular bacteria (see *SI Appendix*, § SV for details). Alternative model closures could offer more accurate angular distributions at large rotational Péclet numbers (albeit at the potential expense of analytic tractability); see, for example, ref. 25. In this work, we only consider the local hydrodynamic effects of fluid-induced rotation and advection terms in Eqs. **3** and **4**, and we assume that the macroscale fluid flow is not affected by the bacterial dynamics. We impose a constant bacterial density, ρ=1, as the inlet condition on Eq. **3**. We discuss the inlet conditions for Eq. **4** in the section below. For now, on the bottom surface at y=0 we impose perfect adhesion i.e. ρ=0; we consider imperfect adhesion in *SI Appendix*, §SVII and discussions of no-flux boundaries are presented in refs. 20 and 26.

In this model framework, the behavior of the collection of bacteria is characterized by two sets of dimensional parameters. The first set are the fluid parameters: the shear rate of the flow $\dot{\gamma }$ and the lengthscale of the flow L, which together set the velocity scale $U=\dot{\gamma }L$. Depending on the application of interest, natural choices for the flow lengthscale L include the radius of the transporting pipe and the size of the viscous fluid boundary layer. We focus on applications in which the fluid speed is significantly faster than the speed of an individual bacterium and, hence, the relative swimming speed is small, $V_{s}\ll 1$. Mathematically, this is a singular limit, and we will exploit this in our following analysis. We note that our analysis and subsequent results are not limited to situations with exact shear flow. Our results will also apply to general flows that can be well approximated by shear flow within the diffusive boundary layer that we identify and analyze below. Finally, for simplicity, in what follows we present analysis of adhesion to a surface in which bacterial motion is restricted to a two-dimensional plane; analysis of bacterial adhesion to a flat surface when the bacteria are free to move in three dimensions is presented in *SI Appendix*, § SVI.

#### 2.2.1. Transport far from the surface.

We start by briefly summarizing the behavior of the suspension in the flow far from the surface, giving full details in *SI Appendix*, § SII. In the next section, we discuss how this behavior changes close to the surface. Far from the surface, the bacterial density described by Eq. **3** is unaffected by adhesion. Since the relative swimming speed is small ($V_{s}\ll 1$), the bacterial distribution is determined here by fluid transport alone:

[6] $$\begin{array}{cc} \frac{D\rho }{\mathrm{Dt}} & =0. \end{array}$$

Eq. **6** is a statement that bacteria move along fluid streamlines. Hence, bacterial density is constant throughout the flow far from the surface and is set by the upstream value ρ=1. In the absence of swimming, the mean bacterial orientation n and the nematic order tensor Q on each streamline are determined by fluid transport, shear alignment, and rotational diffusion alone. In the small swimming limit $V_{s}\ll 1$, Eq. **4** become

[7a] $$\begin{array}{cc} \frac{D(\rho n)}{\mathrm{Dt}} & =\left(\rho In-T\right):\left(\beta E+W\right)-\frac{\rho n}{Pe_{r}}, \end{array}$$

[7b] $$\begin{array}{cc} \frac{D(\rho Q)}{\mathrm{Dt}} & =\beta \rho \left(E(Q+I/2)+(Q+I/2)E\right)+\rho \left(WQ-QW\right)-2\beta G:E-\frac{4\rho Q}{Pe_{r}}. \end{array}$$

Because bacterial density is constant along the streamlines here, we seek solutions to Eq. **7** that are independent of spatial position and time. This means setting the sum of all source terms on the right-hand-sides of Eq. **7** to zero, and solving the resulting algebraic equations. This task is simplified in shear flow as the fluid tensors have the following forms:

[8] $$\begin{array}{c} E=\frac{1}{2}\left(\begin{array}{cc} 0 & 1\\ 1 & 0 \end{array}\right),\;W=\frac{1}{2}\left(\begin{array}{cc} 0 & 1\\ -1 & 0 \end{array}\right). \end{array}$$

The solution of Eq. **7a** that is independent of space and time is n=0, namely that there is no biased swimming direction far from the surface. Similarly, solving Eq. **7b**, we find the following leading-order solution that describes a balance between Jeffery orbit dynamics and rotational diffusion:

[9] $$\begin{array}{c} Q∼\frac{\beta Pe_{r}}{4(16+Pe_{r}^{2})}\left(\begin{array}{cc} Pe_{r} & 4\\ 4 & -Pe_{r} \end{array}\right). \end{array}$$

For circular bacteria (β=0), the nematic order tensor in Eq. **9** is equal to zero, as the angular distribution of circular bacteria is uniform. For elongated bacteria (β>0), the first component of the tensor is positive, reflecting that the bacteria become preferentially aligned parallel to the flow in this case. This is the continuum analogue of the classical Jeffery orbit, in which individual bacteria spend more time oriented parallel to the flow. Note that it is consistent for the bacteria to be preferentially aligned parallel to the flow according to Eq. **9** but for there to be no biased swimming direction, n=0, as the bacteria are equally likely to be facing upstream or downstream and therefore net movement from swimming is zero. Eq. **9** and the solution n=0 provide self-consistent inlet boundary conditions for Q and n (negating the need for an inlet boundary layer), which automatically satisfy Eq. **7** throughout the region away from the surface.

#### 2.2.2. Active Lévêque boundary layer.

**Fig. 2.**
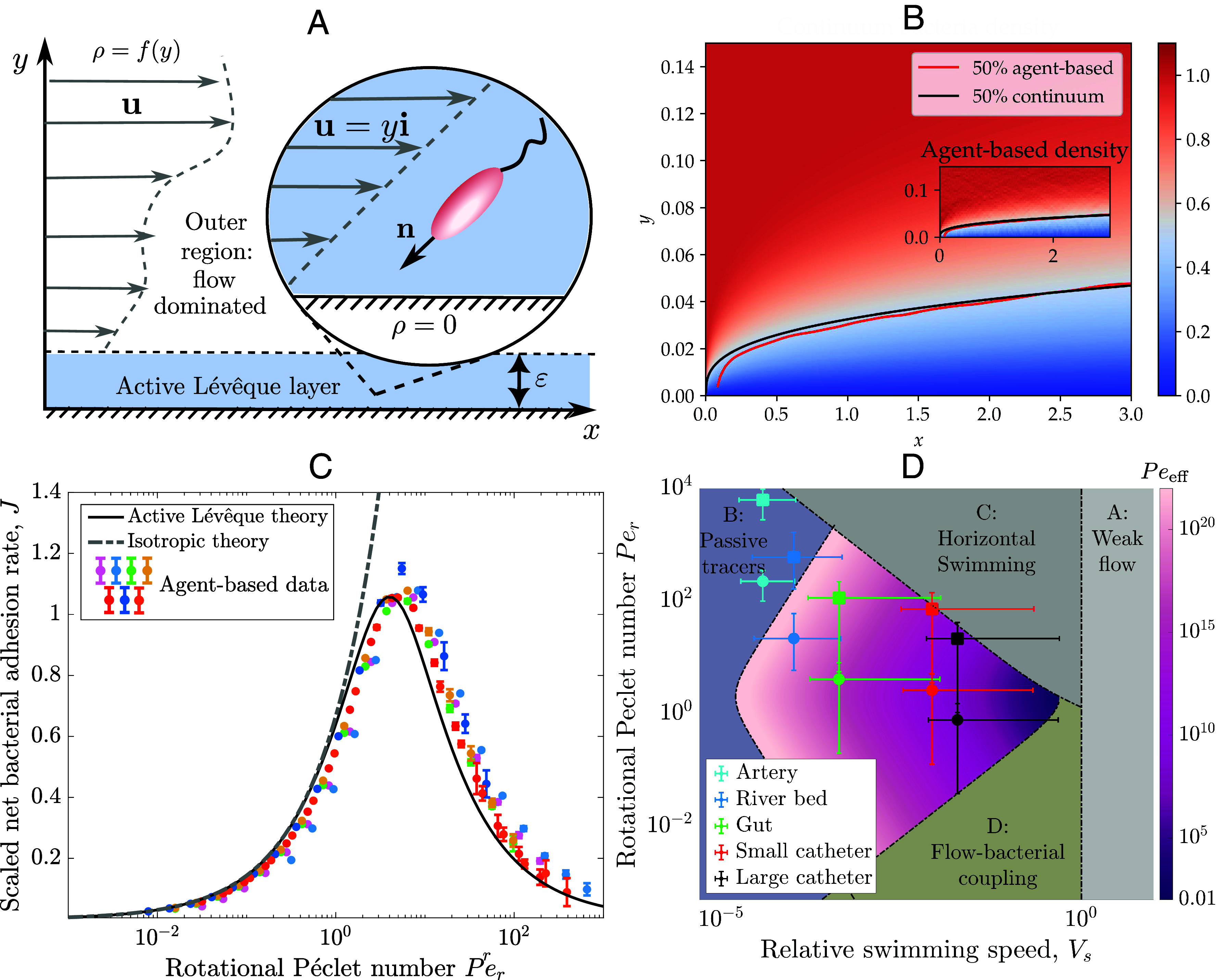
Continuum model for bacterial adhesion in flow. (*A*) Continuum model framework; far from the surface, bacteria are transported by a general laminar flow; in the thin active Lévêque layer the flow is well approximated by shear flow and bacteria density is depleted. (*B*) An analytical solution of the active Lévêque problem for $Pe_{r}=1$, $V_{s}=0.01$, β=0. *Inset*: bacterial density from agent-based simulations with matching contours. Both plots show 50% density contours demonstrating agreement between agent-based density and the continuum model. (*C*) Scaled net bacterial adhesion $\overset{¯}{J}$ (defined in Eq. **17**), is shown as $Pe_{r}$ varies. Agent-based data with colors matching Fig. 1*B* are shown with the SD over individual simulations denoted by the errorbars. Solid and dashed lines show predicted adhesion rates from our active Lévêque theory (using Eq. **15** with $Pe_{\text{eff}}$) and isotropic theory (using Eq. **15** with $Pe_{\text{quies}}$), with no fitting parameters required. (*D*) Value of effective Péclet number $Pe_{\text{eff}}$ (colorbar) is shown for varying $Pe_{r}$ and $V_{s}$ (colorbar). Markers: *E. coli*–circles, *P. aeruginosa*–squares. The assumptions required to calculate our adhesion rate are satisfied for applications with data points in central colored region. Regions A-D define regimes where our analysis does not formally hold—see *Results*.

To predict the adhesion rate of the bacteria in fluid flow, we now consider the behavior of the suspension near the surface. The singular nature of the continuum system we derive means that the behavior of the suspension near the surface is significantly different to the behavior far from the surface. This boundary layer effect arises because adhesion significantly alters the suspension density in a thin region of height ε≪1 near the surface, where we determine *ε* in terms of the system parameters. Our approach generalizes the classic Lévêque theory for passive particles, in which thermal diffusion drives spatial gradients, which in turn lead to adhesion. Here, by contrast, bacterial motility leads to spatial gradients that facilitate adhesion. Hence, we must track the bacterial swimming direction (equivalently, the bacterial orientation) near the surface. Briefly, this involves identifying and analyzing the appropriate asymptotic balance between four processes: fluid transport, bacterial motility, flow-induced rotation, and bacterial rotational diffusion, in the coupled partial differential equation system Eqs. **3** and **4** near the surface. A schematic of the boundary layer structure for the active case is given in Fig. 2*A*. This region is defined by a boundary layer coordinate $\tilde{y}=y/\varepsilon =O\left(1\right)$ and we denote dependent variables in this layer with tildes.

We first note that the steep vertical spatial gradients in the boundary layer mean that the conservation equation Eq. **3** for bacterial density $\tilde{\rho }$ becomes a balance between horizontal transport by the flow and vertical swimming, yielding:

[10] $$\begin{array}{cc} \tilde{y}\frac{\partial \tilde{\rho }}{\partial x}+\frac{V_{s}}{\varepsilon^{2}}\frac{\partial (\tilde{\rho }{\tilde{n}}_{y})}{\partial \tilde{y}} & =0. \end{array}$$

In Eq. **10**, the vertical component of the mean orientation of the bacteria ${\tilde{n}}_{y}$ determines the amount of transport toward the surface. If we are able to write the mean orientation vector $\tilde{n}$ in terms of $\tilde{\rho }$ and the fixed system parameters, we can derive a closed PDE for $\tilde{\rho }$ from Eq. **10**.

We conduct a detailed asymptotic analysis to formally reduce this system in *SI Appendix*, § SIII. Summarizing our analysis, we find that a small anisotropy arises in the mean orientation field due to a balance between i) shear alignment, ii) rotational diffusion, and iii) swimming of bacteria down vertical density gradients. Given our analysis, we can analytically determine the following leading-order solution for the mean orientation of the bacteria, $\tilde{n}=\left({\tilde{n}}_{x},{\tilde{n}}_{y}\right)$:

[11] $$\begin{array}{c} \tilde{\rho }{\tilde{n}}_{y}∼-\frac{4Pe_{r}V_{s}\left(32+\left(2-\beta \right)\left(1-\beta \right)Pe_{r}^{2}\right)}{\varepsilon \left(16+Pe_{r}^{2}\right)\left(16+\left(4-\beta^{2}\right)Pe_{r}^{2}\right)}\frac{\partial \tilde{\rho }}{\partial \tilde{y}},\;\tilde{\rho }{\tilde{n}}_{x}∼-\frac{Pe_{r}^{2}V_{s}\left(64+46\beta +\left(4-\beta^{2}\right)Pe_{r}^{2}\right)}{\varepsilon \left(16+Pe_{r}^{2}\right)\left(16+\left(4-\beta^{2}\right)Pe_{r}^{2}\right)}\frac{\partial \tilde{\rho }}{\partial \tilde{y}}. \end{array}$$

Our analysis shows that the small anisotropy generated within the boundary layer is sufficient to produce enough vertical bacterial transport toward the wall to balance the horizontal transport by the flow in Eq. **10**. Because the anisotropy in the mean orientation field is small, the nematic order tensor remains equal to Eq. **9** and therefore spatially uniform at leading order. Using the vertical component ${\tilde{n}}_{y}$ of Eq. **11** in Eq. **10**, we deduce a (reduced) closed PDE describing the bacterial density near the surface:

[12] $$\begin{array}{cc} \tilde{y}\frac{\partial \tilde{\rho }}{\partial x}-\frac{1}{\varepsilon^{3}Pe_{\text{eff}}}\frac{\partial^{2}\tilde{\rho }}{\partial {\tilde{y}}^{2}} & =0,\;\text{where}\;Pe_{\text{eff}}=\frac{\left(16+Pe_{r}^{2}\right)\left(16+\left(4-\beta^{2}\right)Pe_{r}^{2}\right)}{4Pe_{r}V_{s}^{2}\left(32+\left(2-\beta \right)\left(1-\beta \right)Pe_{r}^{2}\right)}. \end{array}$$

The equivalent result for 3D bacterial motion is given in *SI Appendix*, § SVI. Importantly, Eq. **12** has the same functional form as the classic Lévêque boundary layer problem, so we subsequently refer to it as the active Lévêque problem. We note that (Eq. **12** formally captures the effect of bacterial motility and flow on dispersion near the surface through the effective Péclet number, $Pe_{\text{eff}}$. Seeking a self-consistent balance in Eq. **12** gives an explicit prescription of the boundary layer thickness:

[13] $$\begin{array}{c} \varepsilon \tilde{y}∼{\left(x/Pe_{\text{eff}}\right)}^{1/3}. \end{array}$$

As reassuring consistency checks for the effective Peclet number, Eq. **12**, we note that in the limit $Pe_{r}\to 0$, corresponding to weak flow or strong rotational diffusion, $Pe_{\text{eff}}∼2/V_{s}^{2}Pe_{r}=UL/D_{\text{quies}}=Pe_{\text{quies}}$, which is the classic Péclet number for bacteria when dispersion is controlled by motility alone (16). Additionally, for spherical bacteria (β=0), the effective Péclet number $Pe_{\text{eff}}=\left(4+Pe_{r}^{2}\right)/2Pe_{r}V_{s}^{2},$ which is the 2D equivalent of the $D_{\mathrm{yy}}$ component of the diffusion tensor calculated in ref. 18.

### 2.3. Bacterial Adhesion Rates.

The active Lévêque problem Eq. **12** (accompanied by appropriate boundary and matching conditions) admits a classic similarity solution. This solution is a uniformly accurate description of the density both in the boundary layer and far from the surface

[14] $$\begin{array}{c} \rho \left(x,y\right)=\frac{1}{\Gamma (1/3)}\Gamma \left(\frac{1}{3},\frac{Pe_{\text{eff}}\;y^{3}}{9x}\right), \end{array}$$

which we write in terms of the standard (x,y) coordinates. In Eq. **14**, Γ(a,z) is the lower incomplete gamma function and Γ(a) is the gamma function. The analytic solution Eq. **14** obtained from our boundary layer analysis shows excellent quantitative agreement with the bacteria density calculated from our full agent-based simulations (Fig. 2*B*) everywhere except near x,y=0 (as expected) where there is an additional boundary layer.

The bacterial surface adhesion rate J(x) at a distance *x* from the bacterial source is defined by the vertical swimming flux of bacteria; mathematically this is given by $J\left(x\right)=-V_{s}\rho n_{y}{|}_{y=0}$. Using Eq. **11** and our analytic solution Eq. **14**, we calculate a key result of our study: an analytic expression for the surface adhesion rate of bacteria,

[15] $$\begin{array}{c} J\left(x\right)={\left.-V_{s}\rho n_{y}\right|}_{y=0}=\frac{1}{Pe_{\text{eff}}}{\left.\frac{\partial \rho }{\partial y}\right|}_{y=0}=\frac{3^{1/3}}{\Gamma \left(1/3\right)Pe_{\text{eff}}^{2/3}x^{1/3}}. \end{array}$$

Given the mathematical equivalence noted above, Eq. **15** shares the same functional form as the classic result derived for passive particles in ref. 14, but here with an effective Péclet number that we have calculated explicitly, which captures the interacting effects of bacterial motility, shear-induced alignment, and bacterial shape on adhesion. In dimensional form, this adhesion rate is

[16] $$\begin{array}{c} \hat{J}\left(\hat{x}\right)=\frac{3^{1/3}\rho_{\infty }D_{\text{eff}}^{2/3}{\dot{\gamma }}^{1/3}}{\Gamma \left(1/3\right){\hat{x}}^{1/3}},\;\;D_{\text{eff}}=\frac{4D_{r}V_{s}^{2}\left(32D_{r}^{2}+\left(2-\beta \right)\left(1-\beta \right){\dot{\gamma }}^{2}\right)}{\left(16D_{r}^{2}+\left(4-\beta^{2}\right){\dot{\gamma }}^{2}\right)\left(16D_{r}^{2}+{\dot{\gamma }}^{2}\right)}, \end{array}$$

where $D_{\text{eff}}$ represents the effective diffusion in the boundary layer, $\hat{x}$ is the dimensional distance from the inlet and $\rho_{\infty }$ is the bacterial density at the inlet. Adhesion decreases with distance from the inlet at a rate of ${\hat{x}}^{-1/3}$, and increases with $D_{\text{eff}}^{2/3}$. Elongated bacteria (β>0) have a smaller effective diffusivity and therefore adhere less than spherical bacteria (β=0). We interpret this physically as elongated bacteria preferentially orientating parallel to the flow, reducing the number of bacteria orientated toward the boundary. The reduction in adhesion rate of elongated bacteria is most pronounced at high shear rates because rotational diffusion is unable to significantly reorient bacteria toward the wall. The small effect of Brownian diffusion of the bacteria with diffusion coefficient $D_{T}$, can be included simply, with the resulting effective diffusion coefficient modified to be $D_{\text{eff}}+D_{T}$.

A notable feature of Eq. **16**, compared to the classic Lévêque equivalent, is that $D_{\text{eff}}$ depends on the fluid shear rate in a manner that means the bacterial adhesion rate $\hat{J}$ depends nonmonotonically on the fluid shear rate, with maximal adhesion occurring at a critical shear rate ${\dot{\gamma }}_{\text{crit}}$. In general, ${\dot{\gamma }}_{\text{crit}}$ depends on both rotational diffusivity $D_{r}$ and the shape parameter *β*. For spherical bacteria, we obtain the analytic result ${\dot{\gamma }}_{\text{crit}}=2D_{r}/\sqrt{3}$. For nonspherical bacteria, using parameter values from Table 1, we calculate that maximum adhesion occurs at ${\dot{\gamma }}_{\text{crit}}=$ 1.1 s^−1^ for *E. coli* and ${\dot{\gamma }}_{\text{crit}}=$ 0.039 s^−1^ for *P. aeruginosa*. The nonmonotonic dependence of the adhesion rate on fluid shear rate explains the agent-based findings shown in Fig. 1*B*: adhesion rate $\hat{J}$ scales like $\hat{J}∼{\dot{\gamma }}^{1/3}$ for smaller shear rates until $\dot{\gamma }\approx {\dot{\gamma }}_{\text{crit}}$, then scales with $\hat{J}∼{\dot{\gamma }}^{-1}$. Moreover, our analysis successfully predicts a specific scaling collapse of the scaled net adhesion rate $\overset{¯}{J}$, defined in Eq. **17**, for all agent-based simulations (Fig. 2*C*, see also *SI Appendix*, Fig. S5). The adhesion predicted by our calculated effective diffusivity $D_{\text{eff}}$ correctly shows the maximum adhesion and the decrease in adhesion at high shear (high $Pe_{r}$), in contrast to the adhesion calculated using the isotropic quiescent diffusivity $D_{\text{quies}}=V_{s}^{2}/\left(2D_{r}\right)$ (gray dashed line Fig. 2*C*). While the quiescent approximation works well for low shear rates (low $Pe_{r}$), it does not capture the nonmonotonic nature of the adhesion as the shear rate increases.

The nonmonotonic adhesion rate can be explained physically by examining the orientation and mean swimming speed of the bacteria in the boundary layer, given in Eq. **11**. For small $\dot{\gamma }$ (small $Pe_{r}$), diffusive reorientation is comparatively rapid, and bacteria quickly lose any information about orientational bias arising from density gradients. Therefore, mean vertical swimming is negligibly small, and adhesion is minimal. At moderate shear $\dot{\gamma }$ (moderate $Pe_{r}$), overall swimming persistence is stronger, which increases mean swimming speed and, correspondingly, the adhesion rate. However, at high shear (large $Pe_{r}$), the increased flow rate rotates the bacteria back upstream, reducing the number available to swim into the boundary and lowering adhesion. Our agent-based simulations suggest that this maximum in adhesion rate persists even when the bacteria adhere imperfectly or when they experience long range hydrodynamic surface interactions (27, 28) (*SI Appendix*, §SVII).

The adhesion rate derived in this paper, Eq. **16**, is valid for the typical parameters associated with a wide range of industrial and medical scenarios in which bacterial adhesion and subsequent biofilm formation are problematic. The shear rate near surfaces, also called the wall shear rate, and the flow lengthscales for several such scenarios are presented in Table 2. The motility parameters for common pathogens *E. coli* and *P. aeruginosa* in these scenarios are presented in Table 1. The value of the dimensionless effective Péclet number as a function of dimensionless parameters ($Pe_{r}$, $V_{s}$) is shown in Fig. 2*D* for β=0. Specific examples of the pathogens *E. coli* and *P. aeruginosa* in these industrial and medical settings are shown through the overlaid data points in Fig. 2*D*. For the majority of these applications, the data fall within the colored region in which our formal analysis is valid and, therefore, the system parameters satisfy the requirements to apply our result Eq. **16** to predict adhesion. Furthermore, for all scenarios except *E. coli* adhesion in large catheters, the wall shear rate is greater than the critical shear rate at which maximum adhesion occurs, $\dot{\gamma }>{\dot{\gamma }}_{\text{crit}}$. Our theory therefore predicts that shear-induced reorientation of bacteria reduces adhesion in the industrial and medical scenarios considered and that a prediction based on the classic diffusion coefficient $D_{\text{quies}}$ would overestimate adhesion in these scenarios.

**Table 2.** Wall shear rates and flow lengthscales of industrial and medical systems prone to biofilm formation

Scenario	WSR (1/s)	Lengthscale	Source
Small catheter	0.12 to 5.09	1 mm	(21, 29)
Large catheter	0.038 to 1.51	1.5 mm	(21, 29)
Human small intestine	0.2 to 80	1.25 cm	(30, 31)
Slowly flowing river	5.9 to 21.7	1 cm	(32)
Human coronary artery	100 to 350	2.6 mm	(33, 34)

Our asymptotic theory relies on specific parameter restrictions, which will not hold everywhere in parameter space. We label the regions in which the restrictions do not hold as A–D in Fig. 2*D*, and briefly summarize what happens in each of these regions. In A, the bacterial swimming speed is comparable to the flow speed and, therefore, swimming alters the bacterial density far from the surface, modifying Eqs. **6** and **7**. In B, the boundary layer thickness Eq. **13** is less than the body length of a single bacterium and, therefore, bacteria are transported like ballistic passive tracer particles. In C, shear-induced reorientation is dominant and Eq. **11** does not well describe the bacterial orientation. In this regime, the majority of bacteria are oriented parallel to the surface and there are no longer sufficient numbers of bacteria facing the boundary for our model to accurately capture adhesion. In D, swimming is very strong relative to rotational diffusion, so the nematic order tensor is not spatially uniform and active flows are generated, modifying our result for mean orientation Eq. **11**. Regions A–D are defined in terms of the dimensionless parameters $\left(V_{s},Pe_{r}\right)$ and the boundary layer thickness *ε* as follows. Region A: $V_{s}\geq 1$, Region B: $\varepsilon =5\times 10^{-4}$ (calculated for a bacterium body length of 5 μm and a flow lengthscale L= 1 cm), Region C: $Pe_{r}\geq \varepsilon^{-1}$ and Region D: $V_{s}\leq \varepsilon$. To determine bacterial adhesion in regions A and D, further analysis is required. In regions B and C, bacteria have less opportunity to swim into the boundary, suggesting that the adhesion rate Eq. **16** can provide an upper bound for adhesion even in these regions, thus covering a wider range of relevant flow rates and bacteria species than the formal requirements might suggest.

## 3. Discussion and Conclusion

Using a combination of agent-based and continuum modeling, we have provided a quantitative upper bound for the adhesion rate of bacteria to surfaces, specifically isolating the effects of bacterial motility, bacterial shape, and fluid flow. Our agent-based simulations demonstrate that there is a maximum in adhesion at an intermediate shear rate. Our continuum model and analysis demonstrate that capturing this maximal adhesion rate requires accounting for nonisotropic diffusion, which arises from shear-induced bacterial reorientation. We achieve this by systematically deriving an accurate dispersivity that applies across a range of shear rates. We have found that the classical quiescent dispersivity $D_{\text{quies}}=V_{s}^{2}/2D_{r}$ can only account for adhesion at low flow rates. Our effective dispersivity $D_{\text{eff}}$ applies at shear rates relevant to a wide range of industrial applications.

We have integrated previous bacterial dispersivity results into an active Lévêque boundary layer theory to predict surface adhesion. Our predicted maximal adhesion rate occurs when diffusion parallel to the flow is minimal, which corresponds to intermediate shear rates. Such a reduction in diffusion in the flow direction has been found in studies of bacterial suspensions in nonadhesive pipes (39–41). A reduction in adhesion at high flow rates was also observed in simulations of surface adhesion of spherical bacteria in the presence of a chemotactic gradient (18). In the absence of surface adhesion, motility and shear-induced reorientation have been found to produce active phenomena that differentiate bacterial suspensions from passive mixtures. For example, reorientation has been found to lead to persistent upstream swimming close to nonadhesive surfaces (42, 43). The interaction between these effects in settings with nonuniform shear, such as in channel flows, has also been shown to produce wall accumulation as the density near the center of the pipe is depleted (43, 44). We have demonstrated that both bacterial motility and shear-induced reorientation can drive significant changes in bacterial adhesion, especially in high-flow regimes.

Our work contributes to a general understanding of the complex process of bacterial surface adhesion and colonization. We have isolated the effects of bacterial motility, fluid transport, and shear-induced cell reorientation on adhesion to perfectly absorbing surfaces. To disentangle the specific contribution of these effects, we have not included several further biophysical processes that have been found in experiments to affect bacterial adhesion, such as reversible adhesion, surface chemistry, and cell appendages (5, 7). Our prediction will enable us to isolate the effects of such additional complex processes by comparing measured adhesion rates to our predicted maximal rate. Overall, we have demonstrated how fluid flow and motility can both facilitate and limit attachment in the fundamental problem of bacterial surface adhesion.

## 4. Materials and Methods

### 4.1. Agent-Based Numerical Method.

We simulate the dynamics of bacteria independently in a numerical domain x∈[0,l], y∈[0,H] that is large enough to contain the diffusive boundary layer. We solve Eqs. **1** and **2** in dimensionless form using the explicit stochastic time-stepping scheme Euler–Maruyama with a fixed timestep of $\delta t=10^{-2}$. The bacteria enter the domain at $x=0,y=y_{i}$, where the inlet positions $y_{i}$ are drawn from a probability distribution that describes a uniform suspension advected by shear flow: $p\left(y_{i}\right)=2y_{i}/H^{2}$. The initial inlet orientations are sampled from the numerically calculated diffusive Jeffery orbit distribution (45) (*SI Appendix*, § SIV).

For the spatial density comparison in Fig. 2*B*, we solve the system until t=1,000 using a domain height of H=0.8 and length l=3. At each timestep 100 bacteria enter the domain, which means that we simulate the dynamics of $10^{7}$ bacteria. We record the location of each bacterium over time and form a histogram in (x,y) by calculating the average number of bacteria in each bin over time. We discard the data for t<300 to ensure the boundary layer has reached steady state. We normalize the density field according to the expected number of bacteria in each box (in the absence of motility), as determined by the domain size, the number of boxes and the number of bacteria entering the domain per δt. To measure bacterial adhesion rate across a wide range of motility parameters, we solve the system until t=10,300 with a domain height of H=1.5 and length l=3, simulating approximately $10^{8}$ bacteria. We record the locations of any adhesion events with the boundary that occur within the time interval [300,10,300]. We split this into time intervals of length 2,500 which, since the bacteria are decoupled, is equivalent to carrying out 4 independent simulations over a time window of 2,500. We then calculate the net adhesion occurring over x∈[0,l], and average over each time window to obtain the mean adhesion rate $J_{A}$ and the SD around that mean. To allow comparison between dimensionless agent-based simulations, the net adhesion rate plotted in Fig. 1*B* is in dimensional form as $J_{A}\dot{\gamma }$, which has units of cells/s. The scaled net adhesion rate, $\overset{¯}{J}$, plotted in Fig. 2*C* is defined as the integral of the dimensionless adhesion rate *J* (Eq. **15**) along the surface, scaled by our predicted dependence of the adhesion rate on swimming speed and shape, $S(\beta ,Pe_{r},V_{s})$. We calculate $\overset{¯}{J}$ from the mean net agent-based adhesion rate $J_{A}$ as follows:

[17] $$\begin{array}{c} \overset{¯}{J}=S\left(\beta ,Pe_{r},V_{s}\right)\int_{0}^{l}J\;\text{d}x=\frac{J_{A}S\left(\beta ,Pe_{r},V_{s}\right)}{\rho_{\infty }},S\left(\beta ,Pe_{r},V_{s}\right)=\frac{{\left(16+\left(4-\beta^{2}\right)Pe_{r}^{2}\right)}^{2/3}}{V_{s}^{4/3}{\left(32+\left(2-\beta \right)\left(1-\beta \right)Pe_{r}^{2}\right)}^{2/3}}, \end{array}$$

where the value $\rho_{\infty }$ converts the agent-based adhesion rate, which has units number of bacteria per unit time, to the dimensionless adhesion rate. This is calculated by equating the number of particles arriving per δt in the agent-based simulations with the continuum inlet flux, giving $\rho_{\infty }=8\times 10^{3}$cells μm^−2^. In Eq. **17**, $V_{s}$ and $Pe_{r}$ are calculated from dimensional motility parameters using the shear rate $\dot{\gamma }$ and a flow lengthscale of L=750 μm.

### 4.2. Flow and Bacteria Parameters.

The shear rate (also known as the wall shear rate) and the flow lengthscales for industrial and medical scenarios are presented in Table 2. Dimensional bacterial parameter values for *E. coli* and *P. aeruginosa* are presented in Table 1. These two parameter sets are used to calculate the data points in Fig. 2*D*. We approximate *E. coli*’s run-and-tumble reorientation dynamics as continuous reorientation via rotational diffusion by replacing the rotational diffusion coefficient with the tumble rate. This approximation is valid when the bacteria are dilute and are not subject to external forces (46).

## Supplementary Material

Appendix 01 (PDF)

## Data Availability

The computational tools for agent-based model, data processing scripts, and analytical calculation notebooks are freely available at https://github.com/Edwina-Yeo/bacterial-surface-adhesion (47).
