# Supplementary material for: A shear-induced limit on bacterial surface adhesion in fluid flow
Source: Proc Natl Acad Sci U S A. 2026 Jan 21;123(4):e2516069123. doi: 10.1073/pnas.2516069123 (PMC12846803; doi:10.1073/pnas.2516069123)
Supplement: Supplementary file 1 — Appendix 01 (PDF) [file pnas.2516069123.sapp.pdf]

Supplementary Information: A shear-induced limit on bacterial surface adhesion in fluid flow

Edwina F. Yeo* and Benjamin J. Walker
Department of Mathematics, University College London, London, WC1H 0AY, UK

Philip Pearce
Department of Mathematics, University College London, London, WC1H 0AY, UK and
Institute for the Physics of Living Systems, University College London, London, WC1H 0AY, UK

Mohit P. Dalwadi
Mathematical Institute, University of Oxford, Oxford, OX2 6GG, UK and
Department of Mathematics, University College London, London, WC1H 0AY, UK

SI. DERIVATION AND CLOSURE OF THE CONTINUUM MODEL

In this section we derive Eqs. (3-4) from the agent-based model defined in Eqs. (1-2). We use the model for the transport of a collection of bacteria at the continuum scale following the orientational moment framework from [1], combined with a 2D model closure from [2, 3] to define the higher-order tensors, Eqs. (5). The model is nondimensionalised using a reference velocity scale $\mathcal{U} = \dot{\gamma}\mathcal{L}$ for the external flow, with reference lengthscale $\mathcal{L}$ that is much larger than the size of an individual bacteria, and the timescale of fluid transport $\dot{\gamma}^{-1}$. We start by considering the probability density function $\psi(\boldsymbol{x}, \boldsymbol{s}, t)$, which describes the probability of finding a bacteria at position $\boldsymbol{x}$ with orientation $\boldsymbol{s}$ at time t. This probability density ψ evolves according to the following (dimensionless) Fokker-Planck equation

$$\frac{\partial \psi}{\partial t} + \nabla_{\boldsymbol{x}} \cdot (\boldsymbol{v}\psi) + \hat{\nabla}_{\boldsymbol{s}} \cdot (\boldsymbol{v}_s \psi) - \frac{1}{Pe_r}\hat{\nabla}^2_{\boldsymbol{s}}\psi = 0, \tag{S1}$$

where $\nabla_{\boldsymbol{x}}$ is the gradient operator with respect to spatial variables, $\hat{\nabla}_{\boldsymbol{s}} = (\boldsymbol{I} - \boldsymbol{ss})\nabla_{\boldsymbol{s}}$ is the gradient operator on the unit sphere where $\boldsymbol{I}$ is the identity tensor, $\boldsymbol{ss}$ defines a rank-2 tensor with components $[\boldsymbol{ss}]_{ij} = s_i s_j$ and $\nabla_{\boldsymbol{s}}$ is the gradient operator with respect to orientational variables. The Laplacian operator on the surface of the unit sphere is defined $\hat{\nabla}^2_{\boldsymbol{s}} = \hat{\nabla}_{\boldsymbol{s}} \cdot \hat{\nabla}_{\boldsymbol{s}}$. From left to right the terms in Eq. (S1) are: the evolution of probability in time, the spatial advection of probability, the angular advection of probability and rotational diffusion. The velocity vectors in Eq. (S1) are the dimensionless individual bacterial velocity and angular velocity: $\boldsymbol{v} = \boldsymbol{u} + V_s\boldsymbol{s}$ and $\boldsymbol{v}_s = (\beta\boldsymbol{E} + \boldsymbol{W})\boldsymbol{s}$ with rate of rotation $\boldsymbol{W}$ and strain tensors $\boldsymbol{E}$ defined using the dimensionless velocity field:

$$\boldsymbol{W} = \frac{1}{2}(\nabla\boldsymbol{u} - \nabla\boldsymbol{u}^T), \quad \boldsymbol{E} = \frac{1}{2}(\nabla\boldsymbol{u} + \nabla\boldsymbol{u}^T). \tag{S2}$$

As described in the main text the model has two dimensionless parameters: $V_s = \mathcal{V}_s/\dot{\gamma}\mathcal{L}$, which is the ratio of bacterial swimming speed to the flow speed and $Pe_r = \dot{\gamma}/D_r$, the rotational Péclet number, which is the ratio of fluid rotation to rotational diffusion. We define the density ρ, the mean bacterial orientation $\boldsymbol{n}$ (also known as the polar order parameter) and the nematic order tensor $\boldsymbol{Q}$ of the collection of bacteria as the zeroth, first and second angular moments of the probability ψ as follows

$$\rho(\boldsymbol{x}, t) = \int \psi \,\mathrm{d}\boldsymbol{s}, \quad \boldsymbol{n}(\boldsymbol{x}, t) = \frac{1}{\rho}\int \boldsymbol{s}\psi \,\mathrm{d}\boldsymbol{s}, \quad \boldsymbol{Q}(\boldsymbol{x}, t) = \frac{1}{\rho}\int \left(\boldsymbol{ss} - \frac{\boldsymbol{I}}{2}\right)\psi \,\mathrm{d}\boldsymbol{s}. \tag{S3}$$

The evolution of the density ρ, mean orientation $\boldsymbol{n}$ and nematic order tensor $\boldsymbol{Q}$ can be found by taking the zeroth, first and second moments of Eq. (S1), which gives the following system, as presented in [1, 2]:

$$\frac{D\rho}{Dt} = -V_s \nabla \cdot (\rho\boldsymbol{n}), \tag{S4a}$$

$$\frac{D(\rho\boldsymbol{n})}{Dt} = -V_s\left(\nabla \cdot (\rho\boldsymbol{Q}) + \frac{1}{2}\nabla\rho\right) + (\rho\boldsymbol{In} - \boldsymbol{T}) : (\beta\boldsymbol{E} + \boldsymbol{W}) - \frac{\rho\boldsymbol{n}}{Pe_r}, \tag{S4b}$$

$$\frac{D(\rho\boldsymbol{Q})}{Dt} = -V_s\left(\nabla \cdot \boldsymbol{T} + \frac{\boldsymbol{I}}{2}\nabla \cdot (\rho\boldsymbol{n})\right) + \beta\rho\left(\boldsymbol{E}(\boldsymbol{Q} + \boldsymbol{I}/2) + (\boldsymbol{Q} + \boldsymbol{I}/2)\boldsymbol{E}\right) + \rho(\boldsymbol{WQ} - \boldsymbol{QW}) - 2\beta\boldsymbol{G} : \boldsymbol{E} - \frac{4\rho\boldsymbol{Q}}{Pe_r}. \tag{S4c}$$

* Contact: edwina.yeo.14@ucl.ac.uk

The higher-order moments of $\boldsymbol{s}$, the orientation vector, are: $\boldsymbol{T}$, the third moment and $\boldsymbol{G}$, the fourth moment. These are defined as

$$\boldsymbol{T} = \int \boldsymbol{sss}\psi \, \mathrm{d}\boldsymbol{s}, \qquad \boldsymbol{G} = \int \boldsymbol{ssss}\psi \, \mathrm{d}\boldsymbol{s}. \tag{S5}$$

In this paper we follow [2] and use a truncated angular harmonic closure to define $\boldsymbol{T}$ and $\boldsymbol{G}$. In 2D, this is defined as follows

$$\psi(\boldsymbol{x},\boldsymbol{s},t) = \frac{\rho}{2\pi}\left(1 + 2\boldsymbol{s}\cdot\boldsymbol{n} + 4\boldsymbol{ss}:\boldsymbol{Q}\right), \tag{S6}$$

and is equivalent to approximating the probability ψ using a truncated Fourier series [2]. Consequentially, Eq. (S6) becomes a better approximation the less important higher-order Fourier modes are for ψ, for example if ψ is close to a uniform distribution. We quantify the accuracy of Eq. (S6) in §SV. Under this closure assumption we have the following definitions of the requisite higher-order moments, as stated in [3]:

$$\boldsymbol{T}_{ijk} = \frac{\rho}{4}(\delta_{ij}n_k + \delta_{ik}n_j + \delta_{jk}n_i), \tag{S7}$$

$$\boldsymbol{G}_{ijkl} = \frac{\rho}{8}(\delta_{ij}\delta_{lk} + \delta_{ik}\delta_{jl} + \delta_{il}\delta_{jk}) + \frac{\rho}{6}(\delta_{ij}Q_{lk} + \delta_{ik}Q_{jl} + \delta_{il}Q_{jk} + \delta_{jk}Q_{il} + \delta_{jl}Q_{ik} + \delta_{jk}Q_{ij}). \tag{S8}$$

At higher densities, long-range hydrodynamic effects arising from self generated bacterial flows couple the nematic order tensor to the fluid stress tensor and drive active flows. The relative size of this active stress to the viscous fluid stress is determined by the parameter grouping $g = \sigma N/\mu\dot{\gamma}$, in which σ is the stresslet strength generated by a single bacterium, N is the number of bacteria per unit volume, $\dot{\gamma}$ is a reference shear rate and μ is the fluid viscosity. Using the stresslet value measured for *E. coli* [4] and safe levels of *E. coli* in catheter flow [5] ($\sigma = 7.9\times10^{-19}$N m, $\mu = 10^{-3}$Pa s, $\dot{\gamma} = 100\,\mathrm{s}^{-1}$, $N = 10^{10}/\mathrm{m}^3$) we can estimate $g \approx 10^{-8}$. This demonstrates that bacteria-bacteria hydrodynamic interactions contribute negligibly to the dynamics in the applications of interest, and the fluid flow remains independent of the bacterial dynamics.

SII. DERIVATION OF SOLUTION FAR FROM THE SURFACE

In this section we derive the analytical solutions for the mean orientation vector $\boldsymbol{n}$ and nematic order tensor $\boldsymbol{Q}$ that hold far from the surface. The leading-order equations, which hold far from the surface, are obtained from Eqs. (S4) by removing terms including V_s:

$$\frac{D\rho}{Dt} = 0, \tag{S9a}$$

$$\frac{D(\rho\boldsymbol{n})}{Dt} = (\rho\boldsymbol{In} - \boldsymbol{T}):(\beta\boldsymbol{E} + \boldsymbol{W}) - \frac{\rho\boldsymbol{n}}{Pe_r}, \tag{S9b}$$

$$\frac{D(\rho\boldsymbol{Q})}{Dt} = \beta\rho\left(\boldsymbol{E}(\boldsymbol{Q} + \boldsymbol{I}/2) + (\boldsymbol{Q} + \boldsymbol{I}/2)\boldsymbol{E}\right) + \rho(\boldsymbol{WQ} - \boldsymbol{QW}) - 2\beta\boldsymbol{G}:\boldsymbol{E} - \frac{4\rho\boldsymbol{Q}}{Pe_r}. \tag{S9c}$$

From Eq. (S9a) we find that the bacterial density is constant with $\rho = 1$ far from the surface, which motivates seeking solutions for the mean orientation vector $\boldsymbol{n}$ and nematic order tensor $\boldsymbol{Q}$ that are independent of space and time. Seeking a solution of Eq. (S9b) that is independent of space and time is equivalent to setting the sum of all the terms on the right-hand side equal to the zero vector:

$$(\rho\boldsymbol{In} - \boldsymbol{T}):(\beta\boldsymbol{E} + \boldsymbol{W}) - \frac{\rho\boldsymbol{n}}{Pe_r} = \boldsymbol{0}. \tag{S10}$$

Using the definition of the fluid tensors in simple shear flow:

$$\boldsymbol{E} = \frac{1}{2}\begin{pmatrix}0 & 1\\ 1 & 0\end{pmatrix}, \quad \boldsymbol{W} = \frac{1}{2}\begin{pmatrix}0 & 1\\ -1 & 0\end{pmatrix}, \tag{S11}$$

and after inserting the definition of $\boldsymbol{T}$, Eq. (S7), into Eq. (S10) this gives two algebraic equations for the components of the mean orientation vector $\boldsymbol{n} = (n_x, n_y)$:

$$\left(\frac{1}{2} + \frac{\beta}{4}\right)\rho n_y - \frac{\rho n_x}{Pe_r} = 0, \quad -\left(\frac{1}{2} - \frac{\beta}{4}\right)\rho n_x - \frac{\rho n_y}{Pe_r} = 0. \tag{S12}$$

The only solution to this system for $\beta \in [0,1)$ is $(n_x, n_y) = (0,0)$. Therefore, away from the surface the bacteria have no biased swimming direction. We now derive the nematic order solution, Eq. (9). We proceed in a similar manner, seeking a spatially uniform steady solution to Eq. (S9c), equivalent to setting the sum of all terms on the right-hand-side equal to the zero tensor:

$$\beta\rho\left(\boldsymbol{E}(\boldsymbol{Q}+\boldsymbol{I}/2)+(\boldsymbol{Q}+\boldsymbol{I}/2)\boldsymbol{E}\right)+\rho(\boldsymbol{W}\boldsymbol{Q}-\boldsymbol{Q}\boldsymbol{W})-2\beta\boldsymbol{G}:\boldsymbol{E}-\frac{4\rho\boldsymbol{Q}}{Pe_r}=\boldsymbol{0} \tag{S13}$$

The nematic order tensor is a symmetric rank 2 tensor with three unique components in 2D:

$$\boldsymbol{Q}=\begin{pmatrix} Q_{xx} & Q_{xy} \\ Q_{xy} & Q_{yy} \end{pmatrix}. \tag{S14}$$

Hence, after inserting the fluid tensor solutions Eq. (S11), and the definition of $\boldsymbol{G}$ Eq. (S8), into Eq. (S13) we have the following three algebraic equations for the three independent $\boldsymbol{Q}$-tensor components:

$$\rho Q_{xy}-\frac{4\rho Q_{xx}}{Pe_r}=0,\quad \frac{\beta\rho}{4}+\frac{1}{2}\rho Q_{xx}(\beta-1)+\frac{1}{2}\rho Q_{yy}(\beta+1)-\frac{4\rho Q_{xy}}{Pe_r}=0,\quad -\rho Q_{xy}-\frac{4\rho Q_{yy}}{Pe_r}=0. \tag{S15}$$

The solution of the system Eq. (S15) is:

$$\boldsymbol{Q}=\frac{\beta Pe_r}{4(16+Pe_r^2)}\begin{pmatrix} Pe_r & 4 \\ 4 & -Pe_r \end{pmatrix}, \tag{S16}$$

which describes the angular distribution of bacteria far from the surface. We note that $Q_{xx} > 0$ when $\beta > 0$ is consistent with the bacteria undergoing diffusive Jeffery-orbits. That is, elongated bacteria ($\beta > 0$) are more likely to be orientated parallel to the flow. We demonstrate the accuracy of Eq. (S16) as β and Pe_r vary in comparison to numerical solutions of the Fokker-Planck equation in the §SV.

SIII. DERIVATION OF BOUNDARY LAYER SOLUTION

In this section we derive the boundary layer solution for both the mean orientation $\boldsymbol{n}$, Eq. (11), and the density equation, Eq. (12), that hold close to the surface. To do this we carry out a boundary layer analysis on the full system, Eqs. (S4), identifying and examining a thin boundary layer of height $\varepsilon \ll 1$ on the surface, where ε is to be determined in terms of the system parameters. We define a boundary layer coordinate $\tilde{y} = y/\varepsilon = O(1)$ and denote inner variables with tildes. We then seek a steady solution exploiting $V_s \ll 1$, that bacterial swimming is weak. The boundary layer transformation defines a rescaled gradient operator as follows

$$\tilde{\nabla}=\frac{\partial}{\partial x}\boldsymbol{i}+\frac{1}{\varepsilon}\frac{\partial}{\partial\tilde{y}}\boldsymbol{j}, \tag{S17}$$

where $\boldsymbol{i}$ and $\boldsymbol{j}$ are the unit vectors in the x- and y-directions, respectively. Before presenting the full boundary layer equations it is useful to state the values of the various flux terms on the right-hand-sides of Eqs. (S4b)–(S4c) once they have been rescaled according to Eq. (S17) and using the closure Eq. (S6):

$$\tilde{\nabla}\cdot(\tilde{\rho}\tilde{\boldsymbol{Q}})+\frac{1}{2}\tilde{\nabla}\tilde{\rho}=\begin{pmatrix} \frac{\partial}{\partial x}(\tilde{\rho}\tilde{Q}_{xx})+\frac{1}{\varepsilon}\frac{\partial}{\partial\tilde{y}}(\tilde{\rho}\tilde{Q}_{xy})+\frac{1}{2}\frac{\partial\tilde{\rho}}{\partial x} \\ \frac{\partial}{\partial x}(\tilde{\rho}\tilde{Q}_{xy})+\frac{1}{\varepsilon}\frac{\partial}{\partial\tilde{y}}(\tilde{\rho}\tilde{Q}_{yy})+\frac{1}{2\varepsilon}\frac{\partial\tilde{\rho}}{\partial\tilde{y}} \end{pmatrix}, \tag{S18}$$

$$\tilde{\nabla}\cdot\tilde{\boldsymbol{T}}=\begin{pmatrix} \frac{\partial\tilde{T}_{xxx}}{\partial x}+\frac{1}{\varepsilon}\frac{\partial\tilde{T}_{yxx}}{\partial\tilde{y}} & \frac{\partial\tilde{T}_{xxy}}{\partial x}+\frac{1}{\varepsilon}\frac{\partial\tilde{T}_{yxy}}{\partial\tilde{y}} \\ \frac{\partial\tilde{T}_{xxy}}{\partial x}+\frac{1}{\varepsilon}\frac{\partial\tilde{T}_{xyx}}{\partial\tilde{y}} & \frac{\partial\tilde{T}_{xyy}}{\partial x}+\frac{1}{\varepsilon}\frac{\partial\tilde{T}_{yyy}}{\partial\tilde{y}} \end{pmatrix}=\frac{1}{4}\begin{pmatrix} 3\frac{\partial(\tilde{\rho}\tilde{n}_x)}{\partial x}+\frac{1}{\varepsilon}\frac{\partial(\tilde{\rho}\tilde{n}_y)}{\partial\tilde{y}} & \frac{\partial(\tilde{\rho}\tilde{n}_y)}{\partial x}+\frac{1}{\varepsilon}\frac{\partial(\tilde{\rho}\tilde{n}_x)}{\partial\tilde{y}} \\ \frac{\partial(\tilde{\rho}\tilde{n}_y)}{\partial x}+\frac{1}{\varepsilon}\frac{\partial(\tilde{\rho}\tilde{n}_x)}{\partial\tilde{y}} & \frac{\partial(\tilde{\rho}\tilde{n}_x)}{\partial x}+\frac{3}{\varepsilon}\frac{\partial(\tilde{\rho}\tilde{n}_y)}{\partial\tilde{y}} \end{pmatrix}, \tag{S19}$$

$$\frac{\boldsymbol{I}}{2}\tilde{\nabla}\cdot(\tilde{\rho}\boldsymbol{n})=\frac{1}{2}\left(\frac{\partial(\tilde{\rho}\tilde{n}_x)}{\partial x}+\frac{1}{\varepsilon}\frac{\partial(\tilde{\rho}\tilde{n}_y)}{\partial\tilde{y}}\right)\begin{pmatrix} 1 & 0 \\ 0 & 1 \end{pmatrix}. \tag{S20}$$

The source terms in Eqs. (S4b)–(S4c) which arise from rotation by the flow are unchanged in the boundary layer scaling as they do not include any spatial gradients. Therefore their values are equal to the algebraic expressions presented in the previous section: Eqs. (S12) and (S15).

We can now determine the rescaled steady boundary layer equations. First, the rescaled equation for density $\tilde{\rho}$, derived from Eq. (S4a) using Eq. (S17) is

$$\varepsilon\tilde{y}\frac{\partial\tilde{\rho}}{\partial x}+\frac{V_s}{\varepsilon}\frac{\partial(\tilde{\rho}\tilde{n}_y)}{\partial\tilde{y}}+V_s\frac{\partial(\tilde{\rho}\tilde{n}_x)}{\partial x}=0. \tag{S21}$$

First, examining Eq. (S21) is it clear that the transport of bacteria is determined by the size of the two components of the mean orientation vector $\tilde{n}_x$ and $\tilde{n}_y$. The rescaled equations for the mean orientation vector components $\tilde{n}_x$ and $\tilde{n}_y$, derived from Eq. (S4b) using Eqs. (S17), (S18) and (S12) are as follows

$$\underbrace{\varepsilon\tilde{y}\frac{\partial(\tilde{\rho}\tilde{n}_x)}{\partial x}}_{(iv)}=\underbrace{\left(\frac{1}{2}+\frac{\beta}{4}\right)\tilde{\rho}\tilde{n}_y}_{(i)}-\underbrace{\frac{\tilde{\rho}\tilde{n}_x}{Pe_r}}_{(ii)}-V_s\left(\frac{\partial}{\partial x}(\tilde{\rho}\tilde{Q}_{xx})+\underbrace{\frac{1}{\varepsilon}\frac{\partial}{\partial\tilde{y}}(\tilde{\rho}\tilde{Q}_{xy})}_{(iii)}+\frac{1}{2}\frac{\partial\tilde{\rho}}{\partial x}\right), \tag{S22a}$$

$$\underbrace{\varepsilon\tilde{y}\frac{\partial(\tilde{\rho}\tilde{n}_y)}{\partial x}}_{(iv)}=\underbrace{-\left(\frac{1}{2}-\frac{\beta}{4}\right)\tilde{\rho}\tilde{n}_x}_{(i)}-\underbrace{\frac{\tilde{\rho}\tilde{n}_y}{Pe_r}}_{(ii)}-V_s\left(\frac{\partial}{\partial x}(\tilde{\rho}\tilde{Q}_{xy})+\underbrace{\frac{1}{\varepsilon}\frac{\partial}{\partial\tilde{y}}(\tilde{\rho}\tilde{Q}_{yy})+\frac{1}{2\varepsilon}\frac{\partial\tilde{\rho}}{\partial\tilde{y}}}_{(iii)}\right). \tag{S22b}$$

Examining Eqs. (S22) we can identify five potential physical effects which could determine the mean orientation at leading-order their relative sizes:

(i) shear alignment,

(ii) rotational diffusion,

(iii) swimming of bacteria down vertical gradients of density and angular distribution,

(iv) horizontal transport of oriented swimmers from upstream.

Explicitly, these effects (i-iv) appear Eqs. (S22) at locations we highlight with underbraces. All other terms in Eqs. (S22) are asymptotically sub-dominant so could not contribute at leading-order. A balance between effects (i) and (ii) alone leads to the homogenous solution found in the outer region in Section SII. Therefore other terms from Eqs. (S22) must contribute to explain the emergent effects observed in the agent-based simulations of bacteria moving towards and adhering to the wall. For $V_s \ll 1$, we consider the distinguished limit in which a balance between mechanisms (i), (ii), and (iii) yields

$$\tilde{\rho}|\tilde{\boldsymbol{n}}|\sim\frac{\tilde{\rho}|\tilde{\boldsymbol{n}}|}{Pe_r}\sim\frac{V_s}{\varepsilon}\frac{\partial\tilde{\rho}}{\partial\tilde{y}}\text{ as }\varepsilon\rightarrow 0. \tag{S23}$$

The scaling (S23) means that effect (iv), the horizontal advection in Eqs. (S22), is subdominant and does not contribute at leading-order. Using the scaling (S23) in Eq. (S21) we can now determine how the bacterial density evolves. Since both components of the mean orientation vector are of the same asymptotic order, the dominant balance in Eq. (S21) is between horizontal advection and vertical swimming, which generates the leading-order equation:

$$\varepsilon\tilde{y}\frac{\partial\tilde{\rho}}{\partial x}+\frac{V_s}{\varepsilon}\frac{\partial(\tilde{\rho}\tilde{n}_y)}{\partial\tilde{y}}=0. \tag{S24}$$

This balance between vertical swimming and the weak shear flow tells us that we seek a solution in which there is only a small bias in the bacterial orientation in the boundary layer: $|\tilde{\boldsymbol{n}}|\rightarrow 0$ as $\varepsilon\rightarrow 0$.

Finally, we determine the nematic order tensor $\tilde{\boldsymbol{Q}}$ components $\tilde{Q}_{xx}$, $\tilde{Q}_{xy}$ and $\tilde{Q}_{yy}$. We consider the following rescaled equations

derived from Eq. (S4c) using Eqs. (S17), (S19), (S20) and (S15):

$$\varepsilon\tilde{y}\frac{\partial(\tilde{\rho}\tilde{Q}_{xx})}{\partial x} = -\frac{V_s}{4}\left(5\frac{\partial(\tilde{\rho}\tilde{n}_x)}{\partial x} + \underbrace{\frac{3}{\varepsilon}\frac{\partial(\tilde{\rho}\tilde{n}_y)}{\partial\tilde{y}}}_{(c)}\right) + \underbrace{\tilde{\rho}\tilde{Q}_{xy}}_{(a)} - \underbrace{\frac{4\tilde{\rho}\tilde{Q}_{xx}}{Pe_r}}_{(b)}, \tag{S25a}$$

$$\varepsilon\tilde{y}\frac{\partial(\tilde{\rho}\tilde{Q}_{xy})}{\partial x} = -\frac{V_s}{4}\left(\frac{\partial(\tilde{\rho}\tilde{n}_y)}{\partial x} + \underbrace{\frac{1}{\varepsilon}\frac{\partial(\tilde{\rho}\tilde{n}_x)}{\partial\tilde{y}}}_{(c)}\right) + \underbrace{\frac{\beta\tilde{\rho}}{4} + \frac{1}{2}\tilde{\rho}\tilde{Q}_{xx}(\beta-1) + \frac{1}{2}\tilde{\rho}\tilde{Q}_{yy}(\beta+1)}_{(a)} - \underbrace{\frac{4\tilde{\rho}\tilde{Q}_{xy}}{Pe_r}}_{(b)}, \tag{S25b}$$

$$\varepsilon\tilde{y}\frac{\partial(\tilde{\rho}\tilde{Q}_{yy})}{\partial x} = -\frac{V_s}{4}\left(3\frac{\partial(\tilde{\rho}\tilde{n}_x)}{\partial x} + \underbrace{\frac{5}{\varepsilon}\frac{\partial(\tilde{\rho}\tilde{n}_y)}{\partial\tilde{y}}}_{(c)}\right) - \underbrace{\tilde{\rho}\tilde{Q}_{xy}}_{(a)} - \underbrace{\frac{4\tilde{\rho}\tilde{Q}_{yy}}{Pe_r}}_{(b)}. \tag{S25c}$$

The underbraces denote the three potential mechanisms which could determine the leading-order equation nematic order tensor:

(a) shear alignment,

(b) rotational diffusion,

(c) vertical gradients in the mean orientation vector.

The scaling of the mean orientation vector, (S23), combined with the balance in the density transport equation Eq. (S24), means that effect (c) contributes at $O(V_s)$ and therefore subdominant to effects (a) and (b). We can conclude that the nematic order tensor is determined by shear alignment (a) and rotational diffusion (b) as in the outer region, see §SII. Hence the leading-order solution in the boundary layer is therefore equal to Eq. (S16):

$$\tilde{\boldsymbol{Q}} = \frac{\beta Pe_r}{4(16+Pe_r^2)}\begin{pmatrix} Pe_r & 4 \\ 4 & -Pe_r \end{pmatrix}. \tag{S26}$$

We can then state the leading-order equations for the mean orientation vector components

$$0 = \left(\frac{1}{2}+\frac{\beta}{4}\right)\tilde{\rho}\tilde{n}_y - \frac{\tilde{\rho}\tilde{n}_x}{Pe_r} - \frac{V_s}{\varepsilon}\frac{\partial}{\partial\tilde{y}}(\tilde{\rho}\tilde{Q}_{xy}), \quad 0 = -\left(\frac{1}{2}-\frac{\beta}{4}\right)\tilde{\rho}\tilde{n}_x - \frac{\tilde{\rho}\tilde{n}_y}{Pe_r} - \frac{V_s}{\varepsilon}\frac{\partial}{\partial\tilde{y}}\left(\frac{1}{2}\tilde{\rho} + \tilde{\rho}\tilde{Q}_{yy}\right), \tag{S27}$$

which can be solved algebraically to give:

$$\tilde{\rho}\tilde{n}_x \sim -\frac{2Pe_rV_s(8\tilde{Q}_{xy} + (2+\beta)(1+2\tilde{Q}_{yy})Pe_r)}{\varepsilon(16+(4-\beta^2)Pe_r^2)}\frac{\partial\tilde{\rho}}{\partial\tilde{y}}, \quad \tilde{\rho}\tilde{n}_y \sim -\frac{4Pe_rV_s(2+(\beta-2)Pe_r\tilde{Q}_{xy} + 4\tilde{Q}_{yy})}{\varepsilon(16+(4-\beta^2)Pe_r^2)}\frac{\partial\tilde{\rho}}{\partial\tilde{y}}. \tag{S28}$$

Substituting the nematic order tensor components $\tilde{Q}_{xy}, \tilde{Q}_{yy}$ we have, Eq. (11) :

$$\tilde{\rho}\tilde{n}_x \sim -\frac{Pe_r^2V_s(64+48\beta+(4-\beta^2)Pe_r^2)}{\varepsilon(16+Pe_r^2)(16+(4-\beta^2)Pe_r^2)}\frac{\partial\tilde{\rho}}{\partial\tilde{y}}, \quad \tilde{\rho}\tilde{n}_y \sim -\frac{4Pe_rV_s(32+(2-\beta)(1-\beta)Pe_r^2)}{\varepsilon(16+Pe_r^2)(16+(4-\beta^2)Pe_r^2)}\frac{\partial\tilde{\rho}}{\partial\tilde{y}}, \tag{S29}$$

Finally we can insert solution Eq. (S29) into the equation for bacterial density Eq. (S24) giving an equation in density alone, Eq. (12) :

$$\tilde{y}\frac{\partial\tilde{\rho}}{\partial x} - \frac{1}{\varepsilon^3 Pe_{\text{eff}}}\frac{\partial^2\tilde{\rho}}{\partial\tilde{y}^2} = 0, \qquad \text{where } Pe_{\text{eff}} = \frac{(16+Pe_r^2)(16+(4-\beta^2)Pe_r^2)}{4Pe_rV_s^2(32+(2-\beta)(1-\beta)Pe_r^2)}, \tag{S30}$$

which defines both the effective Péclet number Pe_{eff} and the boundary layer thickness $\varepsilon\tilde{y} \sim (xPe_{\text{eff}})^{1/3}$. For spherical bacteria ($\beta = 0$) the effective Péclet number simplifies to

$$Pe_{\text{eff}} = \frac{(4+Pe_r^2)}{2Pe_rV_s^2}. \tag{S31}$$

SIV. NUMERICAL SOLUTION OF THE FOKKER-PLANCK EQUATION FOR ANGULAR DISTRIBUTION

In the agent-based simulations we initialise the bacteria with orientations sampled from the diffusive Jeffery-orbit distribution $\Psi(\theta)$, where θ defines the angle the orientation vector $\boldsymbol{s}$ makes with the x-axis. This distribution is determined by seeking steady, spatially uniform solutions to the Fokker-Plank equation, Eq. (S1), namely $\Psi(\theta)$ satisfies

$$\frac{\partial}{\partial\theta}(v_\theta\Psi) - \frac{1}{Pe_r}\frac{\partial^2\Psi}{\partial\theta^2} = 0, \quad v_\theta = -\frac{1}{2}(1-\beta\cos(2\theta)), \tag{S32}$$

where v_θ is the bacterium's angular velocity in terms of θ. Eq. (S32) is solved imposing periodicity of Ψ in the domain $\theta \in [0,2\pi)$ and that the integral of the Ψ in the interval $\theta \in [0,2\pi)$ is one. We use a truncated Fourier series solution for Eq. (S32) derived in [6]:

$$\Psi = \sum_{n=0}^{N}(a_n\cos(2n\theta) + b_n\sin(2n\theta)). \tag{S33}$$

The coefficients (a_n, b_n) are defined by a series of $2N$ coupled linear equations:

$$2b_n - \beta(b_{n-1} + b_{n+1}) = \frac{8n}{Pe_r}a_n, \tag{S34a}$$

$$-2a_n + \beta((1+\delta_{k,1})a_{n-1} + a_{n+1}) = \frac{8n}{Pe_r}b_n. \tag{S34b}$$

This system is solved applying $a_{N+1}, b_{N+1} = 0$, $b_0 = 0$, $a_0 = 1/(2\pi)$, the latter condition ensures that the integral of Ψ over $\theta \in [0,2\pi)$ is equal to one. Periodicity of the PDF is ensured by the Fourier series solution. In our agent-based simulations we truncate series (S33) at $N = 30$.

SV. ACCURACY OF CONTINUUM MODEL CLOSURE

In this section we quantify the accuracy of the continuum model closure Eq. (S6). We first compare the $\boldsymbol{Q}$-tensor solution Eq. (S16) to direct calculation of $\boldsymbol{Q}$ using definition Eq. (S3) combined with $\psi = \Psi(\theta)$ from Eqs. (S33)-(S34). Numerical solutions of Eqs. (S33)-(S34) with $N = 30$ demonstrate that, as expected, rotational diffusion smooths the angular distribution of bacteria with $\Psi(\theta)$ approaching a uniform distribution for $Pe_r \to 0$ and approaching the classical Jeffery-orbit distribution for $Pe_r \to \infty$, as shown in Fig. S1a ($\beta = 0.25$) and Fig. S1d ($\beta = 0.88$). Comparison of the components of the $\boldsymbol{Q}$-tensor solution Eq. (S16) with direct calculation of $\boldsymbol{Q}$ from the series solution Eq. (S33) using definition Eq. (S3) are shown in Fig. S1b ($\beta = 0.25$) and Fig. S1e ($\beta = 0.88$). The solution Eq. (S16) is a highly accurate approximation of Ψ for modestly elongated bacteria for all $Pe_r \in [10^{-2}, 100]$ and for highly elongated bacteria at small Pe_r. However, Eq. (S16) fails to capture the distribution quantitively for highly elongated bacteria with large Pe_r, because Ψ cannot be accurately approximated by the truncated continuum model closure Eq. (S6), see Fig. S1e. The accuracy of the harmonic closure, Eq. (S6) for modestly elongated bacteria allows our active Lévêque theory to accurately predict bacterial adhesion according to Eq. (15), see Fig. S1c. However, the inability of the closure to capture the angular distribution of highly elongated bacteria at large Pe_r is reflected in the disagreement between the agent based adhesion and the predicted adhesion using Eq. (15) for $\beta = 0.88$ in Fig. S1f.

A comparison of the $\boldsymbol{Q}$-tensor solution Eq. (S16) with direct calculation of $\boldsymbol{Q}$ from the series solution Eq. (S33) for the full range of Bretherton parameters $\beta \in [0,1)$ is shown in Fig. S2. In the main text we use $\beta \in [0,0.4]$ for which Eq. (S16) is accurate for all rotational Péclet numbers $Pe_r \in [10^{-2}, 100]$. Alternative model closures could offer higher accuracy at capturing the angular distribution at large rotational Péclet numbers. Suitable candidates could be the HL1 and HL2 closures derived asymptotically for high flow in [7], although the authors therein state that these closures are not asymptotically valid for pure shear flow.

SVI. BACTERIAL ADHESION WITH 3D BACTERIAL MOTION

In Results B we present analysis of adhesion to a surface in which bacterial motion is restricted to a two-dimensional plane, namely all bacteria are initialised oriented in an (x,y) plane and rotational diffusion is restricted to 2D. In this section, we derive the effective diffusion coefficient for adhesion of bacteria with 3D motion. The surface is defined by $y = 0$, and the flow remains as planar shear flow: $\boldsymbol{u} = (y,0,0)$. The domain is defined by $x, y > 0$ and the inlet is defined at $x = 0$, $y > 0$. The asymptotic balances derived in §SII and §SIII are preserved in 3D. We now state the key differences and present the 3D adhesion rate. The

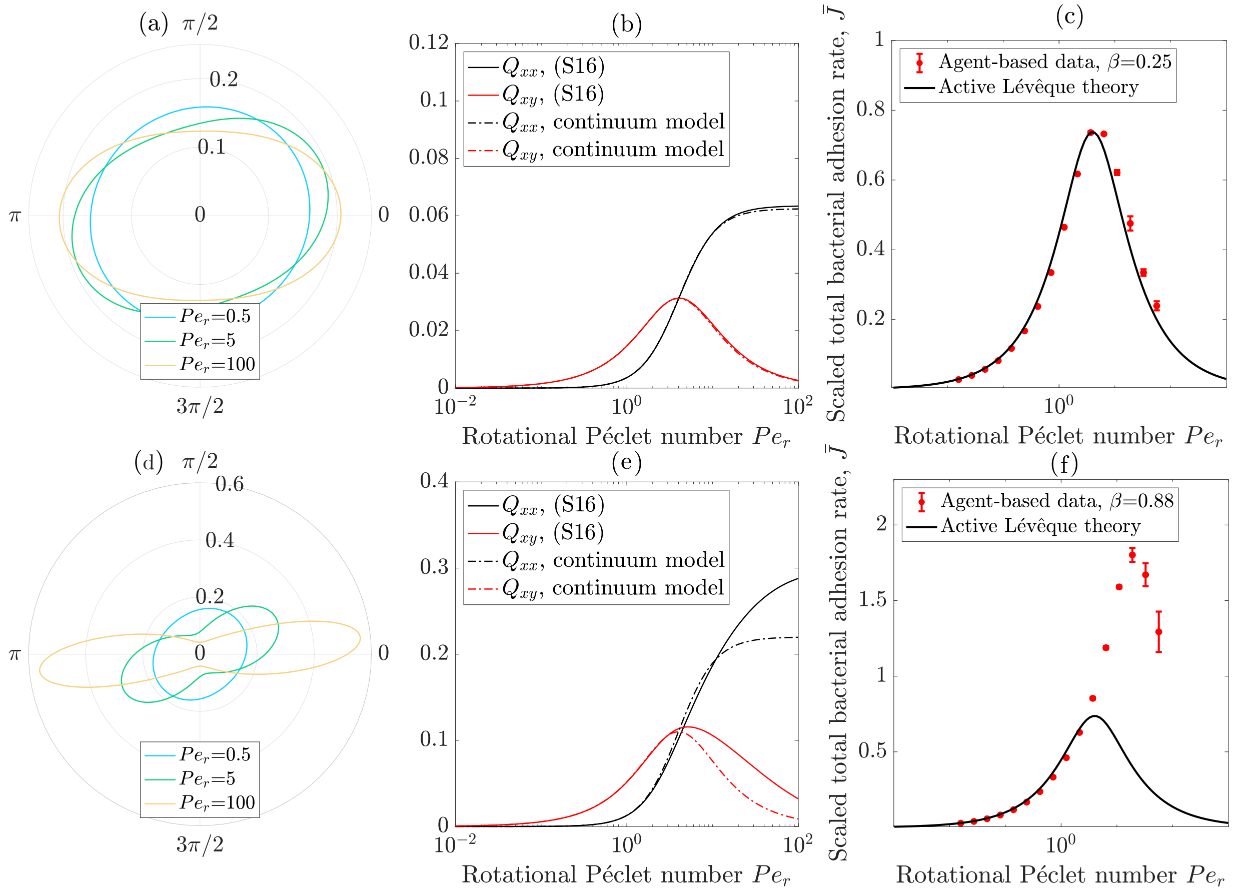

FIG. S1. Angular distribution of the bacteria and the validity of the continuum model for two different bacteria shapes: $\beta = 0.25$ (a–c), $\beta = 0.88$ (d–f). (a) Numerical solution (S33) for $\beta = 0.25$ at varying Pe_r. (b) Comparison of numerical solution (S33) to model solution (S16) with $\beta = 0.25$ shows good agreement for all Pe_r. (c) Agent-based adhesion is well approximated by scaled model adhesion $\bar{J}$ for $\beta = 0.25$. (d) Numerical solution of (S33) for $\beta = 0.88$ at varying Pe_r. (e) Comparison of numerical solution (S33) to model solution (S16) for varying Pe_r ($\beta = 0.88$), shows inaccuracy of the model at large Pe_r. (f) Agent-based adhesion is no longer well approximated by scaled model adhesion $\bar{J}$ for $\beta = 0.88$ at large Pe_r. In all cases we truncate series (S33) at $N = 30$.

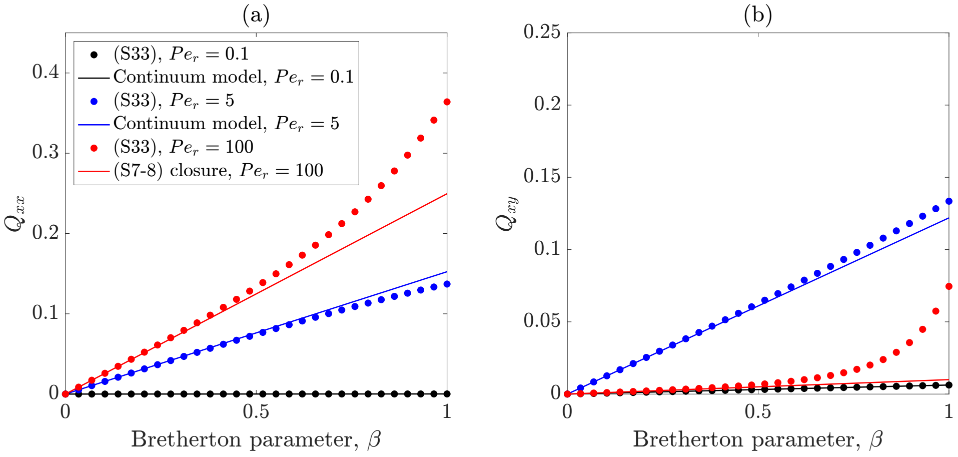

FIG. S2. Comparison of numerical solution (S33) to model solution (S16) for $\beta \in [0, 1)$. In all cases we truncate series (S33) at $N = 30$. (a) Q_{xx} and (b) Q_{xy}.

3D equivalent of the governing equations for ρ, $\boldsymbol{n}$ and $\boldsymbol{Q}$ are given in [1], with the only differences from the 2D version (S4) arising in the factors premultiplying the rotational diffusion terms:

$$\frac{D\rho}{Dt} = -V_s \nabla \cdot (\rho \boldsymbol{n}), \tag{S35}$$

$$\frac{D(\rho \boldsymbol{n})}{Dt} = -V_s \left(\nabla \cdot (\rho \boldsymbol{Q}) + \frac{1}{2} \nabla \rho \right) + (\rho \boldsymbol{I} \boldsymbol{n} - \boldsymbol{T}) : (\beta \boldsymbol{E} + \boldsymbol{W}) - \frac{2\rho \boldsymbol{n}}{Pe_r}, \tag{S36}$$

$$\frac{D(\rho \boldsymbol{Q})}{Dt} = -V_s \left(\nabla \cdot \boldsymbol{T} + \frac{\boldsymbol{I}}{2} \nabla \cdot (\rho \boldsymbol{n}) \right) + \beta \rho \left(\boldsymbol{E}(\boldsymbol{Q} + \boldsymbol{I}/2) + (\boldsymbol{Q} + \boldsymbol{I}/2)\boldsymbol{E} \right) + \rho(\boldsymbol{W}\boldsymbol{Q} - \boldsymbol{Q}\boldsymbol{W}) - 2\beta \boldsymbol{G} : \boldsymbol{E} - \frac{6\rho \boldsymbol{Q}}{Pe_r}. \tag{S37}$$

In 3D, the mean orientation vector is defined $\boldsymbol{n} = (n_x, n_y, n_z)$ and the nematic order tensor is a symmetric rank 2 tensor with six unique components:

$$\boldsymbol{Q} = \begin{pmatrix} Q_{xx} & Q_{xy} & Q_{xz} \\ Q_{xy} & Q_{yy} & Q_{yz} \\ Q_{xz} & Q_{yz} & Q_{zz} \end{pmatrix}. \tag{S38}$$

In 3D, the harmonic closure, (S6), differs slightly, with the tensors $\boldsymbol{T}$ and $\boldsymbol{G}$ now defined as follows (see [1]):

$$\boldsymbol{T}_{ijk} = \frac{\rho}{5}(\delta_{ij} n_k + \delta_{ik} n_j + \delta_{jk} n_i), \tag{S39}$$

$$\boldsymbol{G}_{ijkl} = \frac{\rho}{15}(\delta_{ij}\delta_{lk} + \delta_{ik}\delta_{jl} + \delta_{il}\delta_{jk}) + \frac{\rho}{7}(\delta_{ij} Q_{lk} + \delta_{ik} Q_{jl} + \delta_{il} Q_{jk} + \delta_{jk} Q_{il} + \delta_{jl} Q_{ik} + \delta_{jk} Q_{ij}). \tag{S40}$$

As a result, the analytic solution for the nematic order tensor $\boldsymbol{Q}$ far from the surface is slightly altered:

$$\boldsymbol{Q} = \frac{7\beta Pe_r}{5(1764 + (49 - 3\beta^2)Pe_r^2)} \begin{pmatrix} Pe_r(7+\beta) & 42 & 0 \\ 42 & Pe_r(-7+\beta) & 0 \\ 0 & 0 & -2\beta Pe_r \end{pmatrix}, \tag{S41}$$

Far from the surface, the mean orientation vector is still the zero vector in 3D, $\boldsymbol{n} = \boldsymbol{0}$.

In the boundary layer, the 3D equation for density $\tilde{\rho}$, derived from Eq. (S4a), is

$$\varepsilon \tilde{y} \frac{\partial \tilde{\rho}}{\partial x} + \frac{V_s}{\varepsilon} \frac{\partial (\tilde{\rho} \tilde{n}_y)}{\partial \tilde{y}} + V_s \frac{\partial (\tilde{\rho} \tilde{n}_x)}{\partial x} + V_s \frac{\partial (\tilde{\rho} \tilde{n}_z)}{\partial z} = 0. \tag{S42}$$

From this we can see that the asymptotic balance between shear flow in the x-direction and vertical swimming persists. Following the argument in §SIII, in 3D the nematic-order tensor remains spatially uniform in the boundary layer and is equal to (S41). The 3D version of the mean orientation equations at leading-order, Eq. S27, are:

$$0 = \left(\frac{1}{2} + \frac{3\beta}{10} \right) \tilde{\rho} \tilde{n}_y - \frac{2\tilde{\rho} \tilde{n}_x}{Pe_r} - \frac{V_s}{\varepsilon} \frac{\partial}{\partial \tilde{y}} (\tilde{\rho} \tilde{Q}_{xy}), \tag{S43a}$$

$$0 = -\left(\frac{1}{2} - \frac{3\beta}{10} \right) \tilde{\rho} \tilde{n}_x - \frac{2\tilde{\rho} \tilde{n}_y}{Pe_r} - \frac{V_s}{\varepsilon} \frac{\partial}{\partial \tilde{y}} \left(\frac{1}{3} \tilde{\rho} + \tilde{\rho} \tilde{Q}_{yy} \right), \tag{S43b}$$

$$0 = -\frac{2\tilde{\rho} \tilde{n}_z}{Pe_r} - \frac{V_s}{\varepsilon} \frac{\partial}{\partial \tilde{y}} \left(\tilde{\rho} \tilde{Q}_{yz} \right), \tag{S43c}$$

which have analytic solutions

$$\tilde{\rho} \tilde{n}_x = -\frac{10 Pe_r V_s (60\tilde{Q}_{xy} + (5+3\beta)(1+3\tilde{Q}_{yy}) Pe_r)}{3\varepsilon(400 + (25 - 9\beta^2) Pe_r^2)} \frac{\partial \tilde{\rho}}{\partial \tilde{y}}, \quad \tilde{\rho} \tilde{n}_y = -\frac{10 Pe_r V_s (20 + (3\beta - 5) Pe_r \tilde{Q}_{xy} + 30\tilde{Q}_{yy})}{3\varepsilon(400 + (25 - 9\beta^2) Pe_r^2)} \frac{\partial \tilde{\rho}}{\partial \tilde{y}}, \quad \tilde{n}_z = 0. \tag{S44}$$

Substituting the nematic order tensor components $\tilde{Q}_{xy}, \tilde{Q}_{yy}$ we have:

$$\tilde{\rho} \tilde{n}_x \sim -\frac{88200 Pe_r^2 V_s (1 + \beta + 2(1225 - 3\beta^2(137 - 6\beta)) Pe_r^2)}{3\varepsilon(1764 + (49 - 3\beta^2) Pe_r^2)(400 + (25 - 9\beta^2) Pe_r^2)} \frac{\partial \tilde{\rho}}{\partial \tilde{y}}, \tag{S45}$$

$$\tilde{\rho} \tilde{n}_y \sim -\frac{4 Pe_r V_s (88200 + (2450 - 3(1225 - 461\beta)\beta) Pe_r^2)}{3\varepsilon(1764 + (49 - 3\beta^2) Pe_r^2)(400 + (25 - 9\beta^2) Pe_r^2)} \frac{\partial \tilde{\rho}}{\partial \tilde{y}}, \tag{S46}$$

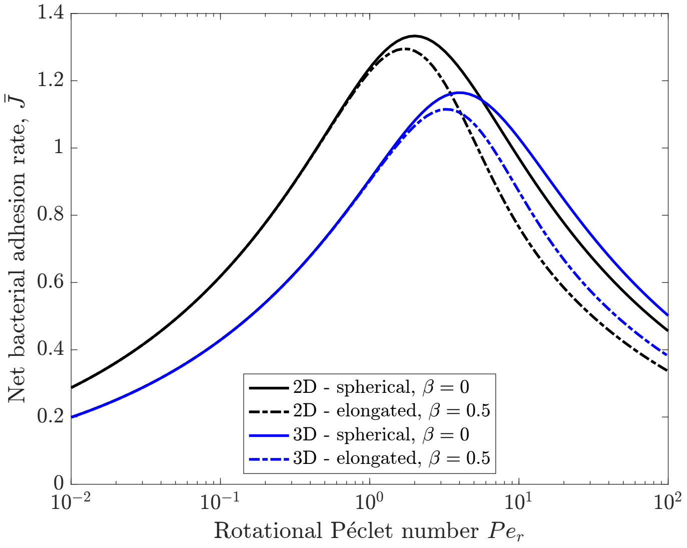

FIG. S3. The predicted net bacterial adhesion rate, (defined in Eq. [17]), is shown as Pe_r varies. In 2D. adhesion is determined by the 2D effective Péclet number, Eq. (S30), and in 3D adhesion rate is determined by the 3D effective Péclet number, Eq. (S47).

Finally, we can insert solution Eq. (S46) into the equation for bacterial density Eq. (S42), giving an equation in density alone:

$$\tilde{y}\frac{\partial \tilde{\rho}}{\partial x} - \frac{1}{\varepsilon^3 Pe_{\text{eff,3D}}}\frac{\partial^2 \tilde{\rho}}{\partial \tilde{y}^2} = 0, \qquad \text{where } Pe_{\text{eff,3D}} = \frac{3(1764 + (49-3\beta^2)Pe_r^2)(400 + (25-9\beta^2)Pe_r^2)}{4Pe_r V_s^2(88200 + (2450 - 3(1225 - 461\beta)\beta)Pe_r^2)}, \tag{S47}$$

which defines the effective Péclet number in 3D, $Pe_{\text{eff,3D}}$. For spherical bacteria ($\beta = 0$), the effective Péclet number simplifies to

$$Pe_{\text{eff,3D}} = \frac{3(16 + Pe_r^2)}{8Pe_r V_s^2}. \tag{S48}$$

In the weak flow limit, as $Pe_r \to 0$, we recover the 3D quiescent diffusion coefficient $Pe_{\text{eff}} = 6/Pe_r V_s^2$. In 3D, the bacterial diffusion is slightly smaller due to the extra degrees of freedom; this leads to a slightly reduced adhesion rate at all flow rates. The adhesion rate is still non-monotonic as a function of shear rate, but with the maximum adhesion rate occurring at a slightly larger flow rate for spherical bacteria in 3D, as shown in Fig. S3.

SVII. AGENT-BASED DATA OF ADHESION WITH ALTERNATIVE WALL INTERACTIONS

In this section we present an exploration of surface adhesion in combination with imperfect adhesion and long range hydrodynamic surface interactions. We model imperfect adhesion through a binding probability $\kappa \in [0,1]$. This probability is assumed to be independent of bacterial orientation, flow rate and bacterial motility parameters. In agent-based simulations, each time a bacteria interacts with the surface the bacteria will bind with probability κ. The remaining bacteria are reflected back into the flow using a total internal reflection condition. Namely, if $y(t_i) < 0$ and $\theta(t_i)$ is the angle relative to the surface then: $(y(t_i), \theta(t_i)) \mapsto (-y(t_i), -\theta(t_i))$. In Fig. S4a we see that overall adhesion is reduced but adhesion rate still obtains a maximum as a function of shear rate when $\kappa < 1$.

We model hydrodynamic surface interactions using the long-range approximation to the force dipole generated by a swimming bacteria. The force dipole generates a resultant flow and rotation on the bacteria given by [8]:

$$\boldsymbol{u}_{\text{wall}} = A\left(\frac{3\sin(2\theta)}{8y^2}, -\frac{3}{8y^2}(1 - 3\sin^2\theta)\right), \quad \dot{\theta}_{\text{wall}} = -\frac{3A\sin(2\theta)}{16y^3}\left(1 + \frac{\beta}{2}(1 + \sin^2\theta)\right). \tag{S49}$$

Here, $A = \alpha/\dot{\gamma}l$ is the dimensionless strength of the hydrodynamic wall-induced effects in comparison to the fluid forces. We take $\alpha = 31.82\,\mu\text{m}^2\,\text{s}^{-1}$, the dimensional flow strength generated by the bacteria, as measured for *E.coli* [4]. For this value of α we find that hydrodynamic surface interactions only significantly alter adhesion rates at high flow rates, see Fig. S4.

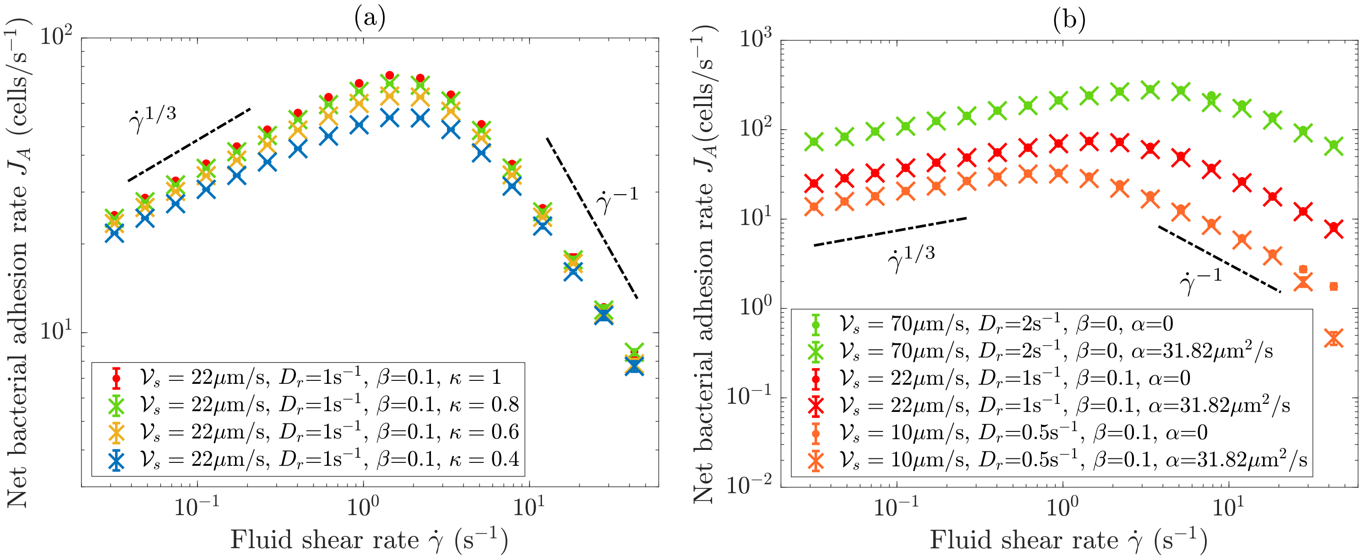

FIG. S4. Bacterial adhesion still obtains a maximum in the presence of additional surface interactions. Agent-based simulations were performed as described in Materials and Methods A; adhesion rates measuring including additional effects are marked with crosses on both plots. To speed up calculation of adhesion rate, we simulate the arrival of $\approx 10^8$ bacteria in parallel, using four repeated intervals from $t \in [0, 2500]$. (a) Including imperfect adhesion with a binding probability κ reduces overall the adhesion rate but a maximum is still obtained. (b) Long-range hydrodynamic surface interactions alter adhesion rates at high flow rates only, with qualitative trends maintained at other flow rates.

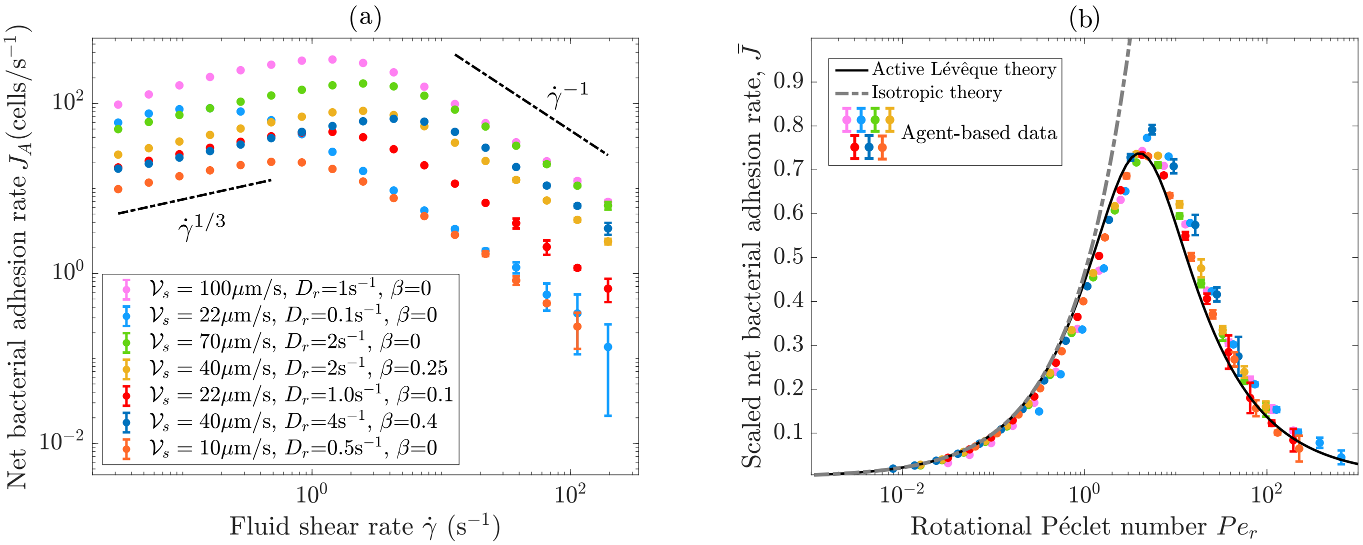

FIG. S5. Net adhesion rate $\bar{J}$ measured in the region $x \in [0.5, l]$ is more accurately predicted by the analytical solution Eq. [14]. Agent-based data adhesion (shown in (a)) collapses more closely to the scaled net bacterial adhesion $\bar{J}$ (defined in Eq. [17]) (shown in (b)). Higher accuracy is gained since the asymptotic solution does not formally hold close to $x = 0$.

[1] D. Saintillan and M. J. Shelley, Theory of active suspensions, in *Complex Fluids in Biological Systems: Experiment, Theory, and Computation*, edited by S. E. Spagnolie (Springer New York, New York, NY, 2015) pp. 319–355.
[2] M. Theillard and D. Saintillan, Computational mean-field modeling of confined active fluids, J. Comput. Phys. **397**, 108841 (2019).
[3] C. Fylling, J. Tamayo, A. Gopinath, and M. Theillard, Multi-population dissolution in confined active fluids, Soft Matter **20**, 1392 (2024).
[4] K. Drescher, J. Dunkel, L. H. Cisneros, S. Ganguly, and R. E. Goldstein, Fluid dynamics and noise in bacterial cell–cell and cell–surface scattering, P.N.A.S. **108**, 10940 (2011).
[5] F. Bull, S. Tavaddod, N. Bommer, M. Perry, C. A. Brackley, and R. J. Allen, Different factors control long-term versus short-term outcomes for bacterial colonisation of a urinary catheter, Nat. Commun. **16**, 3940 (2025).

[6] J. Talbot, C. Antoine, P. Claudin, E. Somfai, and T. Börzsönyi, Exploring noisy Jeffery orbits: A combined Fokker-Planck and Langevin analysis in two and three dimensions, Phys. Rev. E **110**, 044143 (2024).
[7] E. J. Hinch and L. G. Leal, Constitutive equations in suspension mechanics. Part 2. approximate forms for a suspension of rigid particles affected by brownian rotations, J. Fluid Mech. **76**, 187 (1976).
[8] S. E. Spagnolie and E. Lauga, Hydrodynamics of self-propulsion near a boundary: predictions and accuracy of far-field approximations, J. Fluid Mech. **700**, 105–147 (2012).
